# Intermittent fasting positively modulates human gut microbial diversity and ameliorates blood lipid profile

**DOI:** 10.3389/fmicb.2022.922727

**Published:** 2022-08-23

**Authors:** Muhammad Nadeem Khan, Sidra Irshad Khan, Madeeha Ilyas Rana, Arshad Ayyaz, Muhammad Yousaf Khan, Muhammad Imran

**Affiliations:** ^1^Department of Microbiology, Faculty of Biological Sciences, Quaid-I-Azam University, Islamabad, Pakistan; ^2^Department of Biological Sciences, Faculty of Science, University of Calgary, Calgary, AB, Canada; ^3^Department of Pathology, Pakistan Institute of Medical Sciences (PIMS), Islamabad, Pakistan

**Keywords:** human Gut microbiota, intermittent fasting, serum lipid profile, BMI, obesity

## Abstract

**Aim:**

The aim was to evaluate the impact of intermittent fasting (IF) on human body mass index (BMI) and serum lipid profile thorough constructive rectification of gut microbiota.

**Methods and results:**

Fourteen healthy women and thirty-one men were included in the study. Their blood and fecal samples were collected before and at the end of the study. Blood parameters, anthropometric values, and gut microbiology were noted to investigate the impact of intermittent fasting (IF) on human gut microbiota and physiology. Our data revealed that IF reduces the body weight and improves blood lipid profile, such as increasing high-density lipoprotein (HDL) and decreasing total cholesterol, triglycerides, and low- and very low-density lipoprotein levels. IF also decreases culturable aerobic bacterial count and increased fungal count. It was also found that the gut metagenome is altered considerably after IF. The human fecal bacterial diversity exhibited significant changes in decreased overall bacterial population, increased bacterial diversity (alpha diversity), and promoted evenness within the bacterial population at the species level. Anti-inflammatory bacteria *Lactobacillus* and *Bifidobacterium* were favorably increased, while pathogenic bacteria were decreased.

**Conclusion:**

Collectively, these results indicated that IF could improve lipid profile and body weight in humans, and the potential mechanisms might be *via* regulating gut microbiota.

**Significance and impact of the study:**

We demonstrated for the first time that IF improved body weight and blood lipid profile, indicating that IF could mitigate gut microbiota in humans.

## Introduction

Metabolic disorders and associated diseases like diabetes, cardiovascular diseases, inflammatory bowel disease, and various cancers including breast and colorectal cancer are the leading causes of death worldwide (Gibson and Macfarlane, 1995; Berg, 1996; Arumugam et al., 2011; Faulds and Dahlman-Wright, 2012; Rajilić-Stojanović et al., 2015; Blake and Suchodolski, 2016). The diversity and composition of gut microbiota have been identified as an important contributor to metabolic associated disorders, as healthy individuals contain highly diverse gut microbiota, while diseased individuals harbor aberrant and less diverse microbiota in the gut (Friedman, 2009; Le Chatelier et al., 2013; Harmsen and de Goffau, 2016). Moreover, the use of antibiotics in infants predisposes them to unusual weight gain and deposition of adipose tissues later in life, reaffirming that disruptions in gut microbiota composition can lead to the onset of obesity and then metabolic diseases (Cox and Blaser, 2015; Turta and Rautava, 2016). Fecal transplantation from healthy individuals has been proposed to restore normal microbiota. Several studies have demonstrated that fecal transplantation from healthy individuals improved metabolic disorders (cardiovascular disease, type diabetes, and obesity) in diseased individuals (Elinav et al., 2019). Although effective, this approach can lead to a reshuffling of gut microbiome dysbiosis and the emergence of pathobionts (Glauser, 2011; Weil and Hohmann, 2015). Similarly, other available weight loss strategies and modulation of gut microbiota by using antibiotics, prebiotics, and probiotics have many limitations. Like antibiotics kill good microbiota along with pathogens, similarly, it is challenging for probiotic strains to persist in the gut for a longer period. The impact of prebiotic products is also not specific (Druart et al., 2014; Erejuwa et al., 2014). Gonzalez’s group has recently demonstrated in mice that intermittent calorie restriction activates brown adipose tissue and ameliorates obesity by most likely modulating the composition of gut microbiota (Li et al., 2017). In humans, fasting reduces LDL, Vldl, and triglyceride levels, as well as increases HDL levels, as observed in type 2 diabetes patients (Shehab et al., 2012). Intermittent fasting also enhances cardiovascular health, regulates blood pressure by reducing brain natriuretic peptide levels, enhances renal activity (Masoro, 2005; Fontana et al., 2010; Abargouei et al., 2012; Trabelsi et al., 2012; Tiboura et al., 2015), and therefore has become a popular practice around the globe to achieve effective weight loss and improve metabolism. It is still not known how intermittent fasting influences the gut microbiota in humans and whether the regime can support the growth of microbial communities that increase metabolism associated with increased thermogenesis. Also, continuous energy restriction requires a well-defined diet plan that has met with non-compliance issues and adaption. Intermitted fasting or complete abstention from all or selected foods and fluids aiming to restrict food intake for a defined period of time is an easy alternate, is simple to follow, and has shown equal effectiveness in insulin sensitivity, cardiovascular functions, weight loss, and other health biomarkers (Rizza et al., 2014; Mattson et al., 2017).

Here, before and after controlled experimental study in humans was designed to test the effect of intermittent fasting on weight loss, body mass index (BMI), blood lipid profile, C-reactive protein (CRP), and gut microorganisms of obese, normal, and lean but otherwise healthy adult volunteers. We demonstrate that IF improves BMI and reduces triglycerides and low-density lipoprotein levels. Intriguingly, our results also demonstrate that the IF not only decreases gut microbe numbers but also increases their diversity and promotes the growth of microbial species that increase bodily energy metabolism, as identified by the metagenomic analyses.

## Materials and methods

This study was carried out at the Department of Microbiology, Faculty of Biological Sciences, Quaid-i-Azam University, Islamabad, Pakistan.

### Study population and setup

The Intermittent Fasting 91 (16/8 IF) or lean gain method was employed in this study as previously described (Berkhan, 2010). According to this method, participants abstain from foods and liquids for at least 16 h for 26 days. The remaining 8 h of the day serve as the feeding window. During this time, users may eat as many (or few) meals as desired, with the most frequent iteration being two and three meals.

### Approval of the study

The study was approved by the *Bioethical Committee (BEC)* of Quaid-i-Azam University, Islamabad, Pakistan, under protocol number BEC-FBS-QAU2018-109, and all the methods were performed in accordance with the relevant guidelines and regulations advised by the Bioethical Committee (BEC) of Quaid-i-Azam University, Islamabad, Pakistan.

A campaign for the person-to-person meeting was conducted to meet the students (both male and female). The protocol and expected outcomes of the study were orally explained to everyone during the meetings. Students who agreed to participate were enrolled in the study. The age of the volunteers ranged from 18 to 35 years.

**Informed consent for study:** An informed consent was obtained from all the participants for participation in the study, handling of the samples, and data anonymously, and confidentially was also ensured by the research team through this consent (sample of the consent is given in Supplementary material in Supplementary Figure 1). Information about diet and living habits was recorded for all volunteers (given in the Supplementary material in Supplementary Figure 2). Participants were asked to follow the protocol. They were asked to continue their *ad libitum* eating habits during eating widows and live as per routine.

The volunteers who followed the protocol completed the study and provided the required samples (two blood samples and two fecal samples), and their data were included in the current manuscript for the conclusion of the results. The volunteers who were unable to follow the protocol, or complete the study, or were not able to provide the required samples were excluded from the conclusion of the results. Statistical analysis (mean, standard deviation, standard error, paired *t*-test, and agglomerative hierarchical clustering) was performed using Microsoft Excel 2016 and XLSTAT 2019.1.2.

Initially, an equal number of male and female volunteers were selected for the study, but there was a dropout in the samples, and finally, 31 male volunteers and 14 female volunteers were able to complete the study and provide the samples. Furthermore, volunteers were placed in any group of lean, underweight, normal, and overweight or obese where applicable.

Selection criteria included healthy (physically and mentally fit upon looking and feeling), non-smokers, no antimicrobial use (in last 4 weeks), no laxatives use (in last 2 months), no weight reducer use, no diarrhea (in last 1 week), no gastrointestinal tract disease, no exercise more than 10 h/week, and no supplement consumption; those participants provided the samples (two blood samples and two fecal samples), followed the protocol, and completed the study.

### Blood collection and analysis

In total, 10 ml of blood (at once) was collected from the volunteers two times in specified blood collection tubes. One sample was collected 2 days earlier to the study, and the second was collected on the 25th or 26th day of IF (upon convivence). The samples were processed soon after collection, and serum was separated and kept at −20°C until further analysis. Different parameters were recorded from blood analysis. These factors include C-reactive proteins, serum total cholesterol level, serum triglycerides level (TG), high-density lipoproteins (HDL) levels, low-density lipoproteins (LDL) levels, and very low-density lipoproteins (vLDL) levels.

### Fecal sample collection and analysis

The stool samples were collected twice from volunteers. The samples were collected in sterile stool collection containers following the protocols (Wu et al., 2010; Escobar et al., 2014). Soon after collection, each fecal sample was separated into two portions. One portion was stored at −80°C for later use and DNA extraction. In total, 1 g of the fecal sample was taken, diluted in sterile phosphate buffer saline (PBS), taken this as initial dilution, and diluted serially. In total, 100 μl from each dilution was poured and spread on different culture media for the isolation of fecal microorganisms (Castillo et al., 2006). For total microbial aerobic count (TMAC), BHIA (Brain Heart Infusion Agar; Oxoid United Kingdom) media were used. The de Man–Rogosa–Sharpe Agar (MRSA, Oxoid United Kingdom) with 5.4 pH was used to selectively grow the *Lactobacillus spp*. The M17 (Oxoid United Kingdom) was used to grow *Enterococcus, Streptococcus*, and *Lactococcus spp*. MacConkey agar was used to grow the family of *Enterobacteriaceae*, and OGA (Oxytetracycline Glucose Agar; Oxoid United Kingdom) was used for fungal growth. The inoculated plates were incubated at 37°C for 48–72 h (Lim et al., 2004). After incubation, plates were examined for microbial growth. The color, size, and morphology of the grown colonies were noted. The total number of colonies on each plate was counted using a colony counter. An average of 30–300 colonies was used for the actual colony count.

### Extraction of metagenomic DNA from stool

Favor prep Stool DNA isolation mini kit (Favorgen) was used for DNA extraction. Following their designed protocol, 100mg stool was taken in a microtube to which 200mg glass beads were added; frequently, 300 μl of SDE1 buffer and 20 μl of Proteinase K were transferred to the same microtube for disruption of microbial cells. Then, this mixture was vortexed at high speed for 5–7 min and was incubated at 70°C first for 10 min and for an additional 5 min to lyse gram-positive cells. To enhance homogenization, the stool sample was vortexed three times during the period of incubation. The sample was let to cool and added 100 μl of SDE2 buffer. The sample was kept on an ice pack for 5 min and then centrifuged for 5 min at 14,000 rpm. The supernatant was taken and shifted to another microtube with the addition of SDE3, 200 μl buffer. The mixture after mixing was incubated for 2 min at room temperature. The mixture again was centrifuged at said speed for 2 min, and the supernatant was transferred to another sterile microtube. In total, 1 μl of RNase of concentration 100 mg/ml was transferred to the supernatant for the degradation of RNA. After removing the drops from the lid *via* spinning, it was supplemented with 250 μl SDE4 buffer and 250 μl cooled ethanol and mixed by pulse overtaxing. Collection columns were positioned in the collection tube, and the sample was shifted to it. The sample was centrifuged at maximum speed; the column was shifted to another collection tube, and flow-through was discarded; columns were transferred to the next clean collection tube, and flow-through was wasted. Wash buffer (750 μl) was used for washing the impurities by adding them to the column and then centrifuging at maximum speed for 2 min. This step was repeated. For drying the column and avoiding residual contamination, the collection column was centrifuged for 3 min.

The elution tube was taken, and the column was shifted to it. The 70 μl elution buffer was transferred right to the center of the column for elution of the DNA. For complete absorbance of elution buffer into the column, it was centrifuged later to the addition of elution buffer. DNA was eluted after centrifugation and was stored at −20°C for further processing.

### Processing of extracted metagenomic DNA for sequencing

#### Amplification and purification of extracted DNA

PCR was carried out for the amplification of the V4 region of the 16S rRNA gene using forward primer 515F (GTGCCAGCMGCCGCGGTAA) and reverse primer 806R (GGATCACNVGGGTWTCTAAT). The amplification was carried out in triplicates using a volume reaction of 20 μl (Caporaso et al., 2011). HotStar Taq Plus Master Mix (Qiagen, Valencia, CA, United States) and samples were used for the preparation of reaction volume. PCR was performed at these settings: early denaturing, 94°C for 3 min, then tailed by 30 cycles of denaturation at 94°C for 30 sec, annealing at 53°C for 40 s, and finally amplification at 72°C for 1 min. After the entire process, confirmation of the amplification and quality of the gene fragment were tested in 2% agarose gel. Then, as per molecular weight and concentration of DNA, all the samples were joined to each other and were purified by using calibrated Ampure XP beads (Bioscience Corporation, MA, United States).

### Library preparation

Following the Illumina TruSeq DNA library preparation protocol, libraries were prepared from the input of 1 ug of total DNA along with the aforementioned primers *via* the Illumina TruSeq PCR-Free Library Preparation Kit.

### Sequencing

Sequencing was performed at MRDNA sequencing laboratory (Shallowater, TX, United States) using Illumina MiSeq next-generation sequencer. The MiSeq platform performs clonal amplification, genomic DNA sequencing, and data analysis (from assembly to functional classification) with base calling, alignment, variant calling, and reporting in a single run. It performs *de novo* assembly and generates contigs (FASTA) without using a reference genome. It also generates FASTQ files that can be used by third-party tools for analysis. It further aligns the reads against the reference genome and generates a sample report (bam format). Finally, it classifies bacteria according to their 16SrDNA.

### Analysis of the data

All physiochemical data was analysed for statistical significances by using R software (version 3.3.3). One way and two-way ANOVA was used, and results are presented as highly significant (*p* < 0.01) and significant (*p* < 0.05). The *post hoc* test; Student Newman Keuls used to differentiate means after ANOVA significance. In addition to this the two-sample Wilcoxon signed-rank test was used to compare serum lipid profile before and after IF.

Raw sequencing data analysis was done by using MRDNA pipelines (MR DNA, Shallowater, TX, United States). Errors were minimized, and noises were removed. Finally, operational taxonomic units (OTUs) were generated, and chimeras were extruded. OTUs were further defined by clustering at 3% divergence (similarity 97%). Final OTUs were classified taxonomically using BLASTn against a database derived from GreenGenes, RDPII, and RDPI (Desantis et al., 2006). Diversity analysis was done by using QIME2 Microbiome.

## Results

### Impact of intermittent fasting on BMI

**FIGURE 1:**
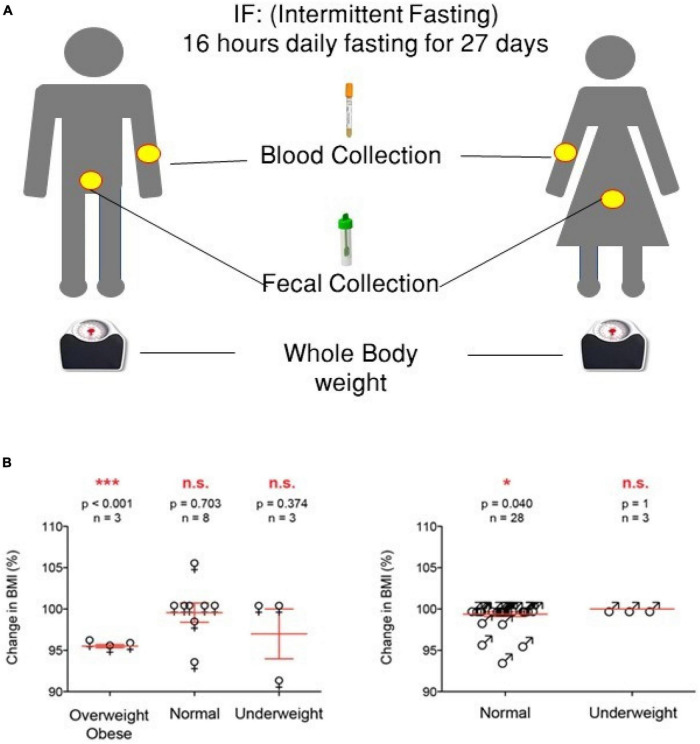
Experimental design of the study and summary of the anthropometric results. **(A)** Experimental design. **(B)** Summary of the anthropometric values [one-way ANOVA was used, highly significant (*p* < 0.01) and significant (*p* < 0.05) values are presented with *** and *, respectively, while non-significant values are presented by N.S].

The impact of IF on weight was personalized, the weight in the majority of normal-weighted male volunteers (25/28; *p* = 0.040) did not change, and only three volunteers reduced their weight, but their BMI remained in the normal range (18.5–24.9; *p* = 1). The weight of underweighted male volunteers (2/3) increased (1–2 kg) (*p* = 0.040). The weight of normal female volunteers (5/16) increased significantly (*p* = 0.001) within the normal BMI range (18.5–24.9), while underweighted female volunteers (2/2) body weight increased and the weight of obese/overweight (3/3 out of total 14) decreased significantly (*p* < 0.001) (Figure 1 and Tables 1, 2).

**TABLE 1:** Detail of the anthropometric values of the male volunteers.

Sample ID	Age (years)	Height (inches)	Weight before study (Kg)	Weight at the end of study (Kg)	BMI before study	BMI at end of study	Fold change BMI	BMI status Before/After study
M1	24	66	65	65	23.13	23.13	NIL	Normal
M2	22	66	60	60	21.35	21.35	NIL	Normal
M3	22	64	65	65	24.59	24.59	NIL	Normal
M4	23	71	57	57	17.52	17.52	NIL	under weight
M5	22	67	57	57	19.68	19.68	NIL	Normal
M6	23	69	64	62	20.83	20.18	NIL	Normal
M7	22	69	65	64	21.16	20.83	0.95	Normal
M8	23	63	54	54	21.08	21.08	NIL	Normal
M9	22	68	70	70	23.46	23.46	NIL	Normal
M10	22	71	68	68	20.90	20.90	NIL	Normal
M11	23	63	56	56	21.86	21.86	NIL	Normal
M12	22	65	54	54	19.80	19.80	NIL	Normal
M13	23	64	53	53	20.05	20.05	NIL	Normal
M14	23	67	58	58	20.02	20.02	NIL	Normal
M15	24	68	66	63	22.12	21.11	0.95	Normal
M16	24	60	75	72	32.28	30.99	0.96	Obese
M17	23	63	50	50	19.52	19.52	NIL	under weight
M18	22	68	63	61	21.11	20.44	0.97	Normal
M19	23	65	54	54	19.80	19.80	NIL	Normal
M20	24	60	53	53	22.81	22.81	NIL	Normal
M21	23	64	57	57	21.56	21.56	NIL	Normal
M22	22	77	57	58	14.89	15.16	1.0	under weight
M23	24	64	63	60	23.83	22.70	0.95	Normal
M24	24	68	68	66	22.79	22.12	0.97	Normal
M25	24	68	67	65	22.45	21.78	0.97	Normal
M26	24	65	58	58	21.27	21.27	NIL	Normal
M27	24	72	68	68	20.32	20.32	NIL	Normal
M28	25	69	70	68	22.78	22.13	0.97	Normal
M29	23	62	53	53	21.36	21.36	NIL	Normal
M30	24	72	65	64	19.43	23.12	1.2	Normal
M31	23	66	56	56	21.34	21.34	NIL	Normal

**TABLE 2:** Detail of the anthropometric values of the female volunteers.

Sample ID	Age (years)	Height (inches)	Weight (Kg) before study	Weight (Kg) at the end of study	BMI before study	BMI at end of study	Fold change BMI	BMI status
F1	25	64	52	52	19.67	19.67	NIL	Normal
F2	23	62	62	62	24.99	24.99	NIL	Underweight
F3	35	64	94	90	35.56	34.05	0.96	Obese II
F4	30	68	115	110	38.54	36.86	0.96	Obese II
F6	24	63	53	63	20.69	24.60	1.19	Normal
F7	23	62	62	59	24.99	23.78	0.95	Overweight/normal
F9	24	63	52	63	20.30	24.60	1.21	
F11	25	64	55	62	20.81	23.45	1.13	Normal
F12	25	65	62	57	22.74	20.90	0.92	Normal
F14	25	62	58	56	23.38	22.57	0.97	Normal
F20	24	64	50	64	18.91	24.21	1.28	Normal
F23	23	63	55	58	21.48	22.64	1.05	Normal
F25	22	62	45	62	18.14	24.99	1.38	Normal
F29	34	63	63	63	24.60	24.60	NIL	Underweight Normal

### Effect of intermittent fasting on acute inflammatory biomarker blood C-reactive protein

Blood C-reactive protein (CRP) was negative for all subject samples (data not shown).

### Effect of Intermittent fasting on serum lipid profile

**FIGURE 2:**
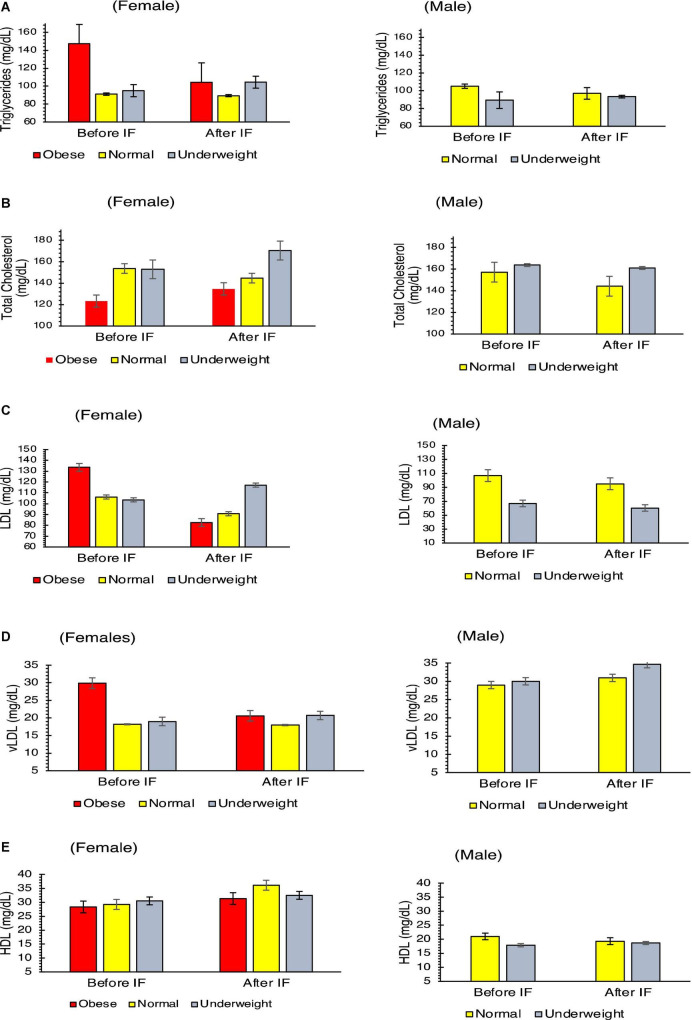
Physiological parameters of blood serum lipid profile of the volunteers before and after intermittent fasting: **(A)** triglycerides, **(B)** total cholesterol, **(C)** low-density lipoproteins, **(D)** very low-density lipoproteins, and **(E)** high-density lipoproteins. Two-sample Wilcoxon signed-rank test was used, all parameters shown a Significant (*p* < 0.05) alteration except vLDL (*p* = 0.117).

By using two-sample Wilcoxon signed-rank test, significant alternation was noticed in total triglycerides (*p* = 0.032), total cholesterol (*p* = 0.008), and low-density lipoprotein (*p* = 0.004) except in very low-density lipoprotein (*p* = 0.117). Total triglycerides decreased from 104 to 100 gm/dl and from 89 to 95 mg/dl (Figure 2A and Supplementary Tables 1, 2), total cholesterol decreased from 152 to 141 mg/dl and from 155 to 133 mg/dl (Figure 2B and Supplementary Tables 1, 2), low-density lipoprotein decreased from 104 to 93 mg/dl and from 102 to 87 mg/dl (Figure 2C and Supplementary Tables 1, 2), and very low-density lipoprotein decreased from 21 to 19 mg/dl and from 19 to 18 mg/dl (Figure 2D and Supplementary Tables 1, 2), while total high-density lipoprotein (HDL) increased from 27 to 28 mg/dl and from 28 to 31 mg/dl (Figure 2E and Supplementary Tables 1, 2) for both the female and male samples, respectively.

### Intermittent fasting alters the diversity and composition of the fecal microbiota

**FIGURE 3:**
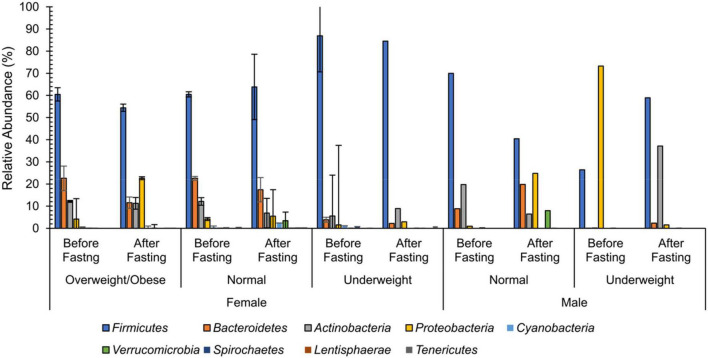
Weighted mean relative abundance of bacteria by 16S rDNA region at the phylum level for female and male volunteers before and after intermittent fasting.

No differences were observed in cultured aerobic bacteria and fungi (Supplementary Figures 1A–E). The impact of intermittent fasting on gut microbiota by culture-independent microbiota was observed. In this assay, ten representative stool samples from individual groups of the human cohort were profiled through 16S DNA sequencing. Reads obtained from sequencing were filtered, low-quality reads were removed, and the remaining reads were classified on phylum, genera, and species level. Similarity tagging was 97% which confirmed the identification of all the species. Richness, α diversity, beta diversity, and dominance of the bacterial population are designed by using the number of reads, OTUs, Shannon, Simpson index, and PCoA (Figure 3 and Table 3). Generally, the total number of OTUs was high in the samples collected before IF while decreasing in samples collected at the end of the study.

**TABLE 3:** Detail of the bacterial diversity of all the samples.

Metagenomic data	Female						Male			
	Obese		Normal		Underweight		Normal		Underweight	
	Before fasting	After fasting	Before fasting	After fasting	Before fasting	After fasting	Before fasting	After fasting	Before fasting	After fasting
No. of Reads	389328	250457	370675	390956	263153	261845	320605	370675	336297	407028
OTUs (Genera)	197	223	212	215	205	200	194	212	193	206
Genera with ≥ 0.1%	82	57	64	83	64	61	21	64	19	52

*All the numbers are given in mean.

At the phylum level, 99% of the intestinal microbiota of this study subject consisted of nine phyla namely *Firmicutes, Bacteroidetes, Actinobacteria, Proteobacteria, Cyanobacteria, Verrucomicrobia, Spirochaetes, Lentisphaerae*, and *Tenericutesirmicutes.* Except for one group (male underweight before fasting), *Firmicutes* were the dominant phylum among all the groups. In the female overweight/obese group, in response to intermittent fasting, *Firmicutes* decreased from the mean percentage of 60.47 to 54.43, *Proteobacteria* increased from 4.17 to 22.61%, *Bacteroidetes* decreased from 22.57 to 11.57%, and *Actinobacteria* decreased from 12.12 to 11.26%. In the female normal group, *Firmicutes* increased from the mean percentage of 60.47 to 63.81, *Proteobacteria* increased from 4.17 to 5.5%, *Bacteroidetes* decreased from 22.57 to 17.41%, and *Actinobacteria* decreased from 12.12 to 6.90%. Similarly, in the female underweight group, *Firmicutes* decreased from the mean of 86.91 to 84.54%, *Proteobacteria* increased from 1.57 to 2.95%, *Bacteroidetes* decreased from 3.86 to 2.18%, and *Actinobacteria* increased from 5.53 to 8.92%.

**FIGURE 4:**
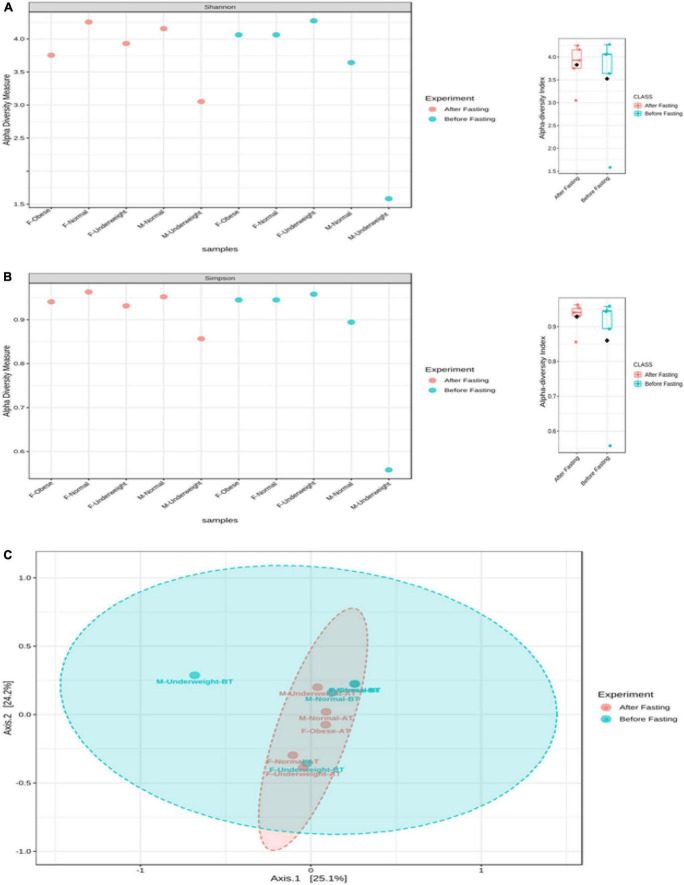
Alpha and beta diversity in volunteer’s intestinal microbiota in response to intermittent fasting. Diversity is shown for each sample and comparative for treatment. **(A)** Alpha diversity (Shannon index), **(B)** alpha diversity (Simpson index), and **(C)** beta diversity (PCoA). The box plot shows diversity for each sample and for experimental group.

The effect of intermittent fasting on male volunteers is unique for both groups. In the male normal group, *Firmicutes* decreased from 69.95 to 40.42% at the end of intermittent fasting, *Proteobacteria* increased from 0.95 to 24.78%, *Bacteroidetes* increased from 8.87 to 19.81%, and *Actinobacteria* decreased from 19.72 to 6.43%. In the male underweight group in response to intermittent fasting, *Firmicutes* increased from the mean percentage of 26.37 to 58.91, *Proteobacteria* decreased from 73.28 to 1.56%, *Bacteroidetes* increased from 0.11 to 2.31%, and *Actinobacteria* increased from 0.19 to 37.14%. Alpha diversity was not significantly altered, while beta diversity was shown significantly altered in obese (Figure 4). The results for the remaining phyla are given in Supplementary Table 3.

**FIGURE 5:**
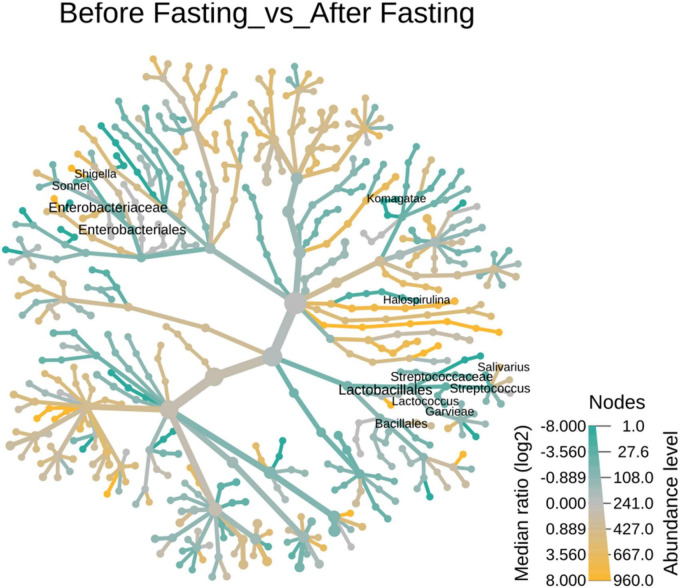
Heat tree of the abundance of taxa at different ranks of the 23 tree hollows in New South Wales, Australia. The size and color of nodes and edges are correlated with the abundance of taxa. The central node is the total of all the other nodes in the tree for each phylum.

The number and type of bacterial genera altered in response to intermittent fasting. Comparative abundance analysis of bacterial diversity involved 365 genera. In total, 82 genera above 0.1% were identified in the fecal microbiota of the female overweight/obese group before intermittent fasting and 57 genera after fasting. In contrast, we found that women of normal BMI had 64 genera before fasting and 83 genera after intermittent fasting. It was also observed that underweight women had 63 genera before fasting and 61 after intermittent fasting. Normal male volunteers had initially 21 genera that changed to 64. Underweight male volunteer’s genera increase from 19 to 52 (Figure 5).

**FIGURE 6:**
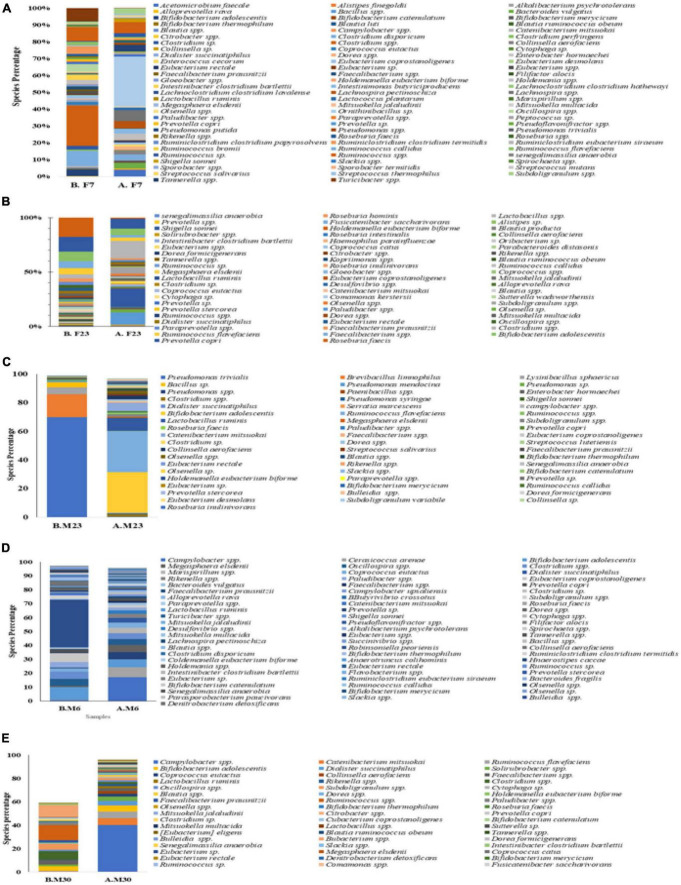
Mean weighted distribution of dominant intestinal bacteria at genus level in response to intermittent fasting: **(A)** obese female volunteers, **(B)** normal female volunteers, **(C)** underweight female volunteers, **(D)** male normal volunteers, and **(E)** male normal volunteers.

In the female overweight/obese group, *Roseburia faecis* was the dominant genera making up 15.34% of all the bacterial population that decreased to 0.97% at the end of the trial. The *Prevotella copri* was the second most dominant group with a percentage of 11.57 that decreased to 7.01% during intermittent fasting. The third most populous genera, *Bifidobacterium adolescentis*, also decreased from 8.41 to 5.80% at the end of the study. The *Faecalibacterium* also decreased from 5.76 to 2.91% at the end of the study. The *Ruminococcaceae*, before fasting, had 4.82% of the overall bacterial population that decreased to 2.91%. Some genera also increased in number when observed at the end of the study trial, for example, *Shigella sonnei* to 16.35% and *Clostridium* spp increased to 14.4% at the end of the trial. The results for other genera are depicted in Figure 6A, and detail is given in Supplementary Table 4.

In the female normal volunteers’ cohort, the *Campylobacter spp.* were the dominant one making up 16.94% of the total fecal microbiota population that decreased to 0.12% at the end of the study trial. The *Clostridiales* also changed from 7.28 to 1.3% at the end of the trial. The *Puniceicoccales* initially was the third major group with *7.23%* of the total, but at the end of the study, it was not detected in the countable form. The *Bifidobacterium adolescentis* also changed in response to intermittent fasting from 5.61 to 0.2%. The *Sphingobacteriales* initially was *5.28%* that later on changed to neglectable count. Dominant genera at the end of the intermittent fasting in female normal volunteers cohort were *Ruminococcus, Eubacteriaceae, Barnesiella, Clostridium*, and *Subdoligranulum* with respective percentages of 12.60, 10.91, 5.44, 5.34, and 4.82. For more results, see Figure 6B and Supplementary Table 5.

Before intermittent fasting, the fecal microbiota of the female underweighted volunteer’s cohort contained *Clostridium spp*. Being the most prominent genera with 18.01% that the end of the trial increased to 24.23%, with *Clostridium disporicum* being the second most prominent genera with 8.63% of total fecal microbiota. The *Ruminococcus* was the third highest in number (7.23%), and the *Erysipelotrichaceae* was the fourth one (6.79%). At the end of the intermittent fasting trial along with *clostridium*, the *Eubacteriaceae* with 6.97% and the *Bifidobacterium adolescentis* with 3.63% were also among the dominant genera. Detail of the other genera is given in Figure 6C and Supplementary Table 6.

**FIGURE 7:**
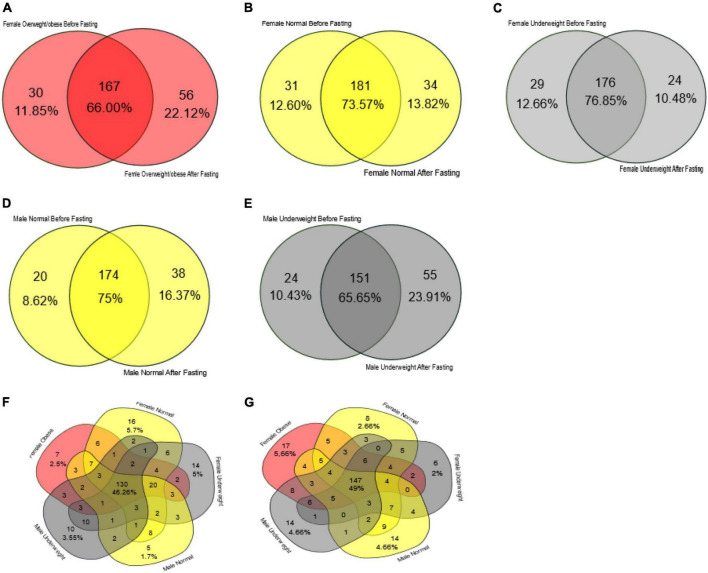
Venn diagram representing core unique and shared intestinal microbiota of female and male volunteers in response to intermittent fasting. **(A)** Obese/overweight female volunteers, **(B)** normal female volunteers, **(C)** underweight female volunteers, **(D)** male normal volunteers, **(E)** male underweight volunteers, **(F)** all volunteers before intermittent fasting, and **(G)** all volunteers after fasting.

In the male normal volunteers’ cohort, the number and type of genera also changed. It was observed that before fasting, the five dominant genera were *Ruminococcus* (35.46%), *Bifidobacterium* (12.31%), *Eubacterium* (7.42%), *Clostridium* (6.33%), and *Oscillospira* (5.04%). Later after the intermittent fasting trial, the five prominent genera were *Campylobacter spp*. (16.94%), *Clostridiales* (728%), *Puniceicoccales* (7.23%), *Bifidobacterium adolescentis* (5.61%), and *Sphingobacteriales* (5.28%). Detail for the remaining genera is given in Figure 7D and Supplementary Table 7.

Similarly, it was observed that the fecal microbiota of underweighted male volunteers has changed in response to intermittent fasting. Before the trial, *Pseudomonas trivialis* (69.47*%), Brevibacillus limnophilus* (16.23%), *Lysinibacillus sphaericus* (4.63), and Bacillus (3.64) were dominating while at end of the trial *Bifidobacterium adolescentis* (28.12%), Ruminococcaceae (25.68), *Lactobacillus ruminis* (9.15%), *Catenibacterium mitsuokai* (5.55%), and Clostridiales (3.58%). Detail for the remaining genera is given in Figure 6E and Supplementary Table 8.

In the female samples, the overweight/obese group had 167 bacterial genera as core, and it makes 66.0% of the total bacterial count. At the start of the study, this group had 30 genera unique while at the end of the study it had 56 genera unique (Figure 7A). The female normal group had 181 bacterial genera as the core that makes up 73.57% of the total bacteria present in the fecal sample. Initially, it had 31 bacterial genera which increased to 34 at the end of the study (Figure 7B). The female underweight group had 176 (76.85%) bacterial genera as the core; at the start of the trial, 29 unique genera were associated with this group later on reduced to 24 (Figure 7C). In the male samples, the normal group had 174 bacterial genera as a core that makes up almost 75% of the total bacterial flora. In the remaining 25%, initially, it had eight unique genera, while at the end of the study, there was an increase in unique species and it had 38 genera “Figure 7D.” The male underweight group had 151 species as core, making 65.65% of the total bacterial flora. Initially, there were 24 bacterial genera unique, which increased to 55 later on at the end of the study (Figure 7E). The number of core bacterial genera in all the groups before intermittent fasting was 130 (46.26%) (Figure 7F) and after intermittent fasting was 147 (49%) (Figure 7G).

## Discussion

Human physiology is significantly impacted by trillions of gut microbiota having nine million non-redundant genes (Hooper and Gordon, 2001; Yang et al., 2009). Dysbiosis of the gut microbiome causes a variety of metabolic disorders that leads to chronic inflammations which are depicted by overweight and obesity as phenotypic outcomes (Vuksan et al., 2016). Body weight is a physical indicator of metabolic diseases (Swanton et al., 2007). Different strategies like the use of probiotics (Fooks and Gibson, 2002), prebiotics, fecal transplantation (Delzenne et al., 2013), and the use of antibiotics are being tried to restore the gut microbiota to normal. In this context, fasting is considered an ancient therapy (Handschin, 2016). Numerous research studies find out that IF mostly results in weight loss and improvement of blood profile (Aloui et al., 2016).

In this study, it was found that IF has an impact on body mass indexes, blood lipid profiles, and gut microbial profiles. It was found that observing IF reduced the BMI of obese/overweight individuals, and there was a slight reduction of BMI in normal men, while no change was found in normal women. Surprisingly, weight increase was observed in underweight women. These results show that this IF regimen tends to normalize the total body mass without its unwarranted depletion in healthy individuals. Reduction of body weight by IF has been reported (Adlouni et al., 1997; Ley et al., 2006; Kul et al., 2014); however, normalization of body weight is reported for the first time. The number of *Bifidobacteria* and *Lactobacillus* increased in all five groups, and it is well established that reduced body weight is linked to an increasing trend in gut microbial diversity, and overweight or obesity is linked to reduced gut bacterial diversity (Ley et al., 2006). In our study, the weight reduction was found linked to an increasing trend in bacterial diversity, while a decreasing trend in bacterial diversity was observed especially in women those gained weights. In an earlier report, the impact of IF on a female subject showed an increase in weight was due to the use of energy-dense diets and less physical activities during the fasting days (Sadeghirad et al., 2014; Savitri et al., 2016). However, the impact of IF is not gender-specific, when tested in the mice model (Piotrowska et al., 2016). Abdelgadir in 2015 conducted a study on three different nations and reported that people using normal diets with physical activities have improved lipid profiles after IF while people eating more or energy-rich diets or doing no or limited physical activities have no change in their lipid profile at the end of study (Abdelgadir et al., 2015). Yeoh et al. (2015) performed a study in 2015 on 29 volunteers in Singapore, and at the end of the study, he concluded that weight was reduced, and lipid profile was also improved in volunteers who were taking routine diets and doing physical activities (Yeoh et al., 2015).

It was also observed that the IF trial lowered the level of total cholesterol but retained it in the normal recommended range (<200 mg/dl). The level of triglycerides decreased in 24/31 male volunteers while increased in 5/31 male volunteers. In the same way, the level of triglycerides is retained in the normal range (< 150 mg/dl). The level of low-density lipoprotein (LDL) decreased in 20/31 male volunteers and increased in 6/31 male volunteers in the same recommended ranges of <100 mg/dl. The level of high-density lipoprotein (HDL) increased in 24/31 and decreased in 3/31 male volunteers but in the normal range (>40 mg/dl), and the level of very low-density lipoproteins decreased in 22/31 male volunteers and increased in 6/31 male volunteers. The total cholesterol level decreased in all female volunteers. The total triglycerides decreased in 11/14 female volunteers and increased only in three volunteers, but within the normal recommended range, low-density lipoproteins decreased in 12/14 female volunteers, and HDL increased in 13/14 female volunteers. IF lowered total aerobic bacterial count in all the male and female volunteers when checked through conventional culturing methods. The number of total *Lactococcus* and total *Lactobacillus*, when assessed through culturing method, seen increased at the end of the IF trial in both male and female volunteers.

**FIGURE 8:**
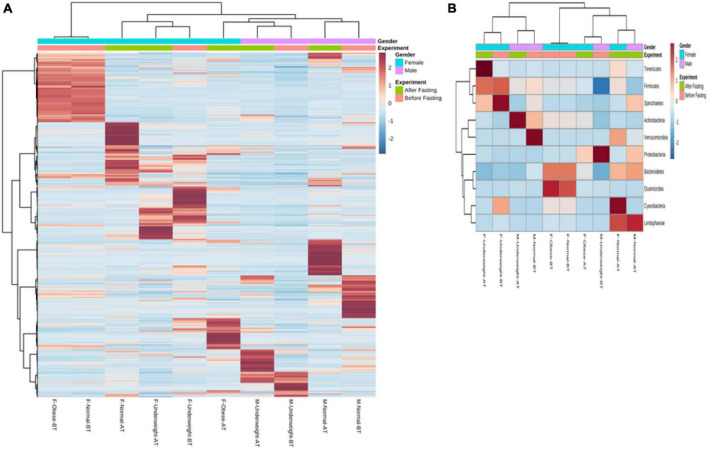
Hierarchically clustered heatmap of the bacterial distribution of different communities. Double hierarchical dendrogram shows the bacterial distribution. The bacterial phylogenetic tree was calculated using the neighbor-joining method, and the relationship among samples was determined by Bray–Curtis distance and the complete clustering method. The heatmap plot depicts the relative percentage of each bacterial phylum (variables clustering on the vertical axis) within each sample group (horizon-axis clustering). The relative values for bacterial phylum are indicated by the color intensity with the legend indicated at the bottom of the figure.

Recently, a study established the link between gut bacterial diversity and lipid blood profile, which showed that fasting sugar level, total triglycerides, total cholesterol, and low-density lipoproteins are associated with reduced gut bacterial diversity as compared to the control cohort (Rebolledo et al., 2017). Another study conducted on Finnish-men also showed that elevated blood lipids are associated with increased gut bacterial diversity (Org et al., 2017). Yang et al. (2017), in 2017, conducted a study on pigs, which showed that increased blood lipid levels are associated with altered bacterial diversity while *Lactobacillus* is negatively correlated with the blood lipid profile (Yang et al., 2017; Liu et al., 2018). The *in vitro* cholesterol assimilation activity of gut microbiota isolated from healthy individuals has been confirmed by different researchers recently, and the presence of bile salt hydrolase *bsh* genes is reported in *Lactobacillus* and *Bifidobacteria*, mostly used for cholesterol assimilation activities (Pereira and Gibson, 2002; Begley et al., 2006; Kim et al., 2009, 2013). These bacteria are known for anti-inflammatory activities, and inulin is the key component that favorably increases the number of *Bifidobacteria spp* (de Souza et al., 2017). These findings favored our results where all the samples carried *Lactobacillus spp* while their lipid profile was also positively improved. It has been demonstrated that overweight individuals carry a high microbial load in their intestines when compared with normal ones (Mokkala et al., 2016). Another study showed that increased bacterial richness is associated with parasite-positive guts (Andersen et al., 2016). It was explained by Maine Margo that increased diversity in the intestine is considered a healthy flora. They have more potential to degrade and metabolize diverse nutrient components (Maine and Kelly, 2016). Therefore, the reduction in bacterial richness and increase in diversity confirm a positive alteration in the intestinal bacteria. Changes were seen in the percentages of bacterial phylum at the end of the study (Figure 8). Normal weight male group had a higher percentage of *Proteobacteria* 73.28% which decreased to 1.56% by intermittent fasting. *Firmicutes* were initially 26% while increased to 58% making the highest portion of all the phylum at the end of the study. Increase in *Actinobacteria* from 0.19 to 37.14% was observed at the end of the study. Male underweighted group before IF had the highest *Firmicutes* of 69.95% and later decreased to 40.42%. The *Actinobacteria* also reduced from 19.72 to 6.43%, while *Proteobacteria* increased from 0.95 to 24.78%, *Bacteroides* from 8.87 to 19.81%, and *Verrucomirobia* from 0.21 to 7.98% at the end of the study. In female underweighted group, *Firmicutes* and *Bacteroides* decreased from 63.18 to 38.55% and 17.41 to 5.43%, respectively, while *Proteobacteria* increased from 5.52 to 41.96% and *Actinobacteria* from 6.90 to 13.97% at the end of the study. Female overweight/obese group before IF carried high *Firmicutes* than *Bacteroides* and low *Proteobacteria*. At the end of the study, *Firmicutes* and *Bacteriodes* were decreased and *Proteobacteria* was increased up to 11%. In female normal group, no significant changes at phylum level were observed. It has been concluded that too high and too low *Proteobacteria* are signs of dysbiosis. A minor percentage of *Proteobacteria* (<12%) is known healthy one at phylum level (Shin et al., 2015).

At the genera level, before IF, it was seen that the normal-weighted male group had a high percentage of *Pseudomonas triavilis*, that is, 69%. This bacterium is isolated from grasses and is kept in risk group one, and it may cause infection in humans (Söhngen et al., 2013). Later, at the end of the study, it decreased to 0.04%. The *Brevibacillus limnophilus* was in the second number (4%) followed by Bacillus spp. At the end of the study, the number of *Ruminococcus flavefaciens* increased to 28%, *Bifidobacterium adolescentis* to 28%, and *Lactobacillus rumins* to 9%. A study conducted by Cucchi et al. (2013) showed that the fecal material of vegetarians has a high number of *Ruminococcus, Lactobacillus*, and *Bifidobacteria* sp. Participants consumed a vegetable-rich diet during the study period. The *Ruminococcus flavefaciens* are fiber digesting bacteria mainly involved in interactive digestion and acquisition of the nutrients in the intestine (Cucchi et al., 2013). Ejtahed et al. (2012), investigated and found that increase in the number of Bifidobacterium is responsible for blood cholesterol lowering. In another study the effect of energy restriction on body weight and gut microbiota was evaluated. It was concluded that an increase in the number of *Bifidobacterium sp* and *lactobacillus spp* is associated with weight loss (Santacruz et al., 2009).

A normal-weighted male group at the end of the IF study had 5% *Catenibacterium mitsoukai* of the total bacterial population. This species is heterofermentative and has a broad range of substrate utilization, thus providing more nutrients to their host (Kageyama and Benno, 2000). Before IF, there were no detectable species of *Collinsella aerofaciens*, but later on, it was observed that they make up 2% of all the total bacterial population present in the gut. The *Collinsella aerofaciens* was associated with the prevention and cure of irritable bowel diseases (Kassinen et al., 2007). This group carried 2% *Subdoligranulum spp*. These species have been used for the treatment of type 2 diabetes (Wang and Jia, 2016). Less number of *Dorea spp* was observed, and it is known that an increased number of *Dorea spp* can contribute to the development of irritable bowel disease (Rajilić-Stojanović et al., 2015). These findings present an improvement in the health status of the intestine and the whole host. In the male underweighted group, evenness and bacterial diversity were increased after the trial. It carried more percentage of *Campylobacter spp*, that is, 16% of the total bacterial population. Studies have shown that *Campylobacter spp* are normally present in animals and poultry, and their count goes high in warmer months. Not all *Campylobacter spp* are pathogenic to humans (Keener et al., 2004). The *Campylobacter* spp. have shown the colonization of the large intestine (Saint-Cyr et al., 2016). This study was performed in warmer months, while the volunteers were consuming poultry meat as a part of their routine diet. Hence, the high concentration of *Campylobacter sp* in the fecal samples of the volunteer’s cohort might be the competitive exclusion of these undesired microorganisms by the intestinal flora. The *Megasphaera elsdenii* were 5% of the total bacterial population. This bacterium is responsible for the treatment of metabolic acidosis (Marx et al., 2011). The *Oscillospira* spp. were decreased in the groups. According to a study, a decrease in *Oscillospira spp* is considered anti-obesity signature bacteria (Walters et al., 2014). Before IF, overweight/obese female group carried a high number of *Roseburia spp* (17%) and *Prevotell copri* (16%). At the end of IF, they were decreased to 1 and 8%, respectively. Increased *Roseburia* spp. are associated with gall stone formation (Tamanai-Shacoori et al., 2017), while increased *Prevotella copri* is associated with autoimmune diseases (Campbell, 2014).

**Strength of the study:** Blood lipid profile was improved and modulated gut microbiota even though the volunteers were taking their *ad libitum* diets during the eating windows of the experiment and were living their routine life. No dietary or living habits were advised to be changed or restricted, but, in many gut modulation studies, experimental subjects are passed through dietary restriction or changing living styles. Recently, a study conducted on a mice model for the improvement of blood lipid profile and modulation of gut microbiota kept the experimental mice restricted to a diet having the main portion of isoquercetin and inulin (Tan et al., 2018). In another study, antibiotics were administered in the diet for the improvement of blood lipid profile and modulation of gut microbiota (Rune et al., 2016). We have conducted our experimental trial on the human population, and it reflects the actual outcome and can be used as a reference for further studies, while in many cases, such studies are performed on mice model. There is a difference in the gut microbiota of humans and mice which affects the outcome of any experimental trial. Recently, the gut microbiota of mice and humans was compared using large datasets, and it was found that both the gut microbiota are similar at phylum level but have large variations at the species level (Xiao et al., 2015). The core microbiota of mice is small, while the core of human gut microbiota is large (Kostic et al., 2013). It was found that 85% of genera that are present in mice are not present in humans (Nguyen et al., 2015), while, when compared at the gene level, only 4% of genes were found in mice microbiota (Xiao et al., 2015). The gut microbiota of mice can be changed by the diet within 1 week, while human gut microbiota does not change so quickly (Wang and Jia, 2016). The difference between the results of women and men may be due to the fasting days, and women were exempted to fast during their menstrual cycle. The number of fasting days is not defined yet.

**Limitation of the study:** The number of male and female samples was not equal and thus may have an effect on the results. This may have influenced the results.

## Data availability statement

The datasets presented in this study can be found in online repositories. The names of the repository/repositories and accession number(s) can be found below: https://www.ncbi.nlm.nih.gov/bioproject/PRJNA477600, PRJNA477600.

## Ethics statement

The studies involving human participants were reviewed and approved by BEC-FBS-QAU2018-109. The patients/participants provided their written informed consent to participate in this study.

## Author contributions

MI and MIR conceived the study and experimental designing. MI supervised the study. AA and MI analyzed the data and critically revised the manuscript. MYK contributed to sample analysis. MNK and SIK conducted experimental work and drafted the manuscript. All authors contributed to the preparation of the final manuscript.

## Supplementary material

Supplementary Figure 1 Informed consent form for participants.

Supplementary Figure 2 Diet and living habits of Participants.

Supplementary Figure 3 Impact of intermittent fasting on culturable aerobic bacteria and fungi from fecal material of participants.

Supplementary Table 1 Impact of intermittent fasting of serum lipid profile of male participants.

Supplementary Table 2 Impact of intermittent fasting of serum lipid profile of female participants.

Supplementary Table 3 Impact of intermittent fasting of gut microbiota at phylum level of participants.

Supplementary Table 4 Impact of intermittent fasting of gut microbiota at genera level of obese female participants.

Supplementary Table 5 Impact of intermittent fasting of gut microbiota at genera level of normal weight female participants.

Supplementary Table 6 Impact of intermittent fasting of gut microbiota at genera level of under-weight female participants.

Supplementary Table 7 Impact of intermittent fasting of gut microbiota at genera level of normal weight male participants.

Supplementary Table 8 Impact of intermittent fasting of gut microbiota at genera level of under-weight male participants.
