# Supplementary figures and images for: Intermittent fasting positively modulates human gut microbial diversity and ameliorates blood lipid profile
Source: Front Microbiol. 2022 Aug 23;13:922727. doi: 10.3389/fmicb.2022.922727 (PMC9445987; doi:10.3389/fmicb.2022.922727)

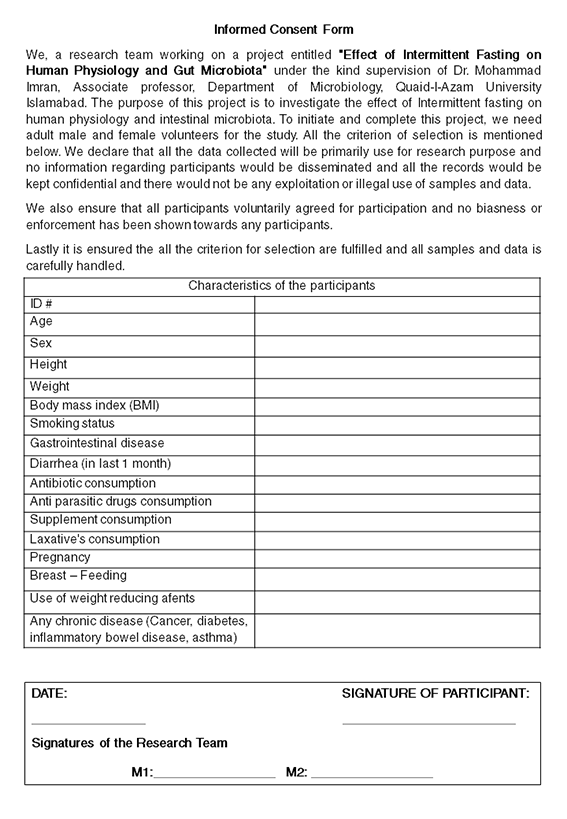

Supplement: Supplementary Figure 1 — Informed consent form for participants. [file Image_1.tif]

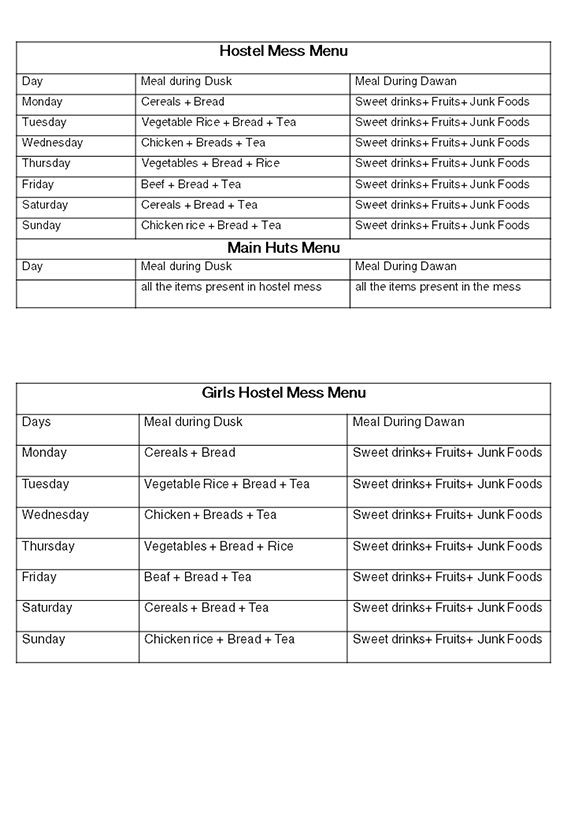

Supplement: Supplementary Figure 2 — Diet and living habits of Participants. [file Image_2.tif]

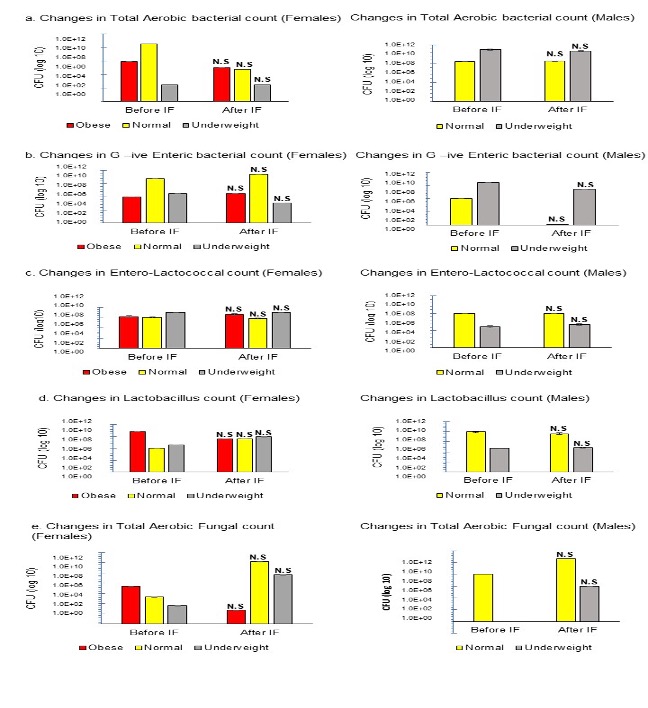

Supplement: Supplementary Figure 3 — Impact of intermittent fasting on culturable aerobic bacteria and fungi from fecal material of participants. [file Image_3.jpeg]
