# Supplementary material for: Intermittent fasting positively modulates human gut microbial diversity and ameliorates blood lipid profile
Source: Front Microbiol. 2022 Aug 23;13:922727. doi: 10.3389/fmicb.2022.922727 (PMC9445987; doi:10.3389/fmicb.2022.922727)
Supplement: Supplementary Table 1 — Impact of intermittent fasting of serum lipid profile of male participants. [file Table_1.docx]

| Sample id | Before TC (mg/dL) | After TC  (mg/dL) | before Tg  (mg/dL) | After Tg  (mg/dL) | Before HDL  (mg/dL) | After HDL  (mg/dL) | Before LDL  (mg/dL) | After LDL  (mg/dL) | Before vLDL  (mg/dL) | After vLDL  (mg/dL) |
| --- | --- | --- | --- | --- | --- | --- | --- | --- | --- | --- |
| M1 | 102 | 97 | 65 | 68 | 22 | 23 | 67 | 60.4 | 13 | 13.6 |
| M2 | 103 | 105 | 91 | 90 | 21 | 21 | 63.8 | 66 | 18.2 | 18 |
| M3 | 160 | 133 | 95 | 97 | 28 | 26 | 113 | 87.6 | 19 | 19.4 |
| M4 | 105 | 103 | 86 | 84 | 21 | 23 | 66.8 | 63.2 | 17.2 | 16.8 |
| M5 | 171 | 152 | 109 | 108 | 30 | 32 | 119.2 | 98.4 | 21.8 | 21.6 |
| M6 | 153 | 103 | 81 | 73 | 25 | 26 | 111.8 | 62.4 | 16.2 | 14.6 |
| M7 | 156 | 164 | 105 | 113 | 30 | 29 | 105 | 112.4 | 21 | 22.6 |
| M8 | 258 | 243 | 160 | 129 | 40 | 41 | 186 | 176.2 | 32 | 25.6 |
| M9 | 228 | 219 | 125 | 110 | 40 | 41 | 163 | 156 | 25 | 22 |
| M10 | 105 | 105 | 84 | 84 | 23 | 23 | 65.2 | 65.2 | 16.8 | 16.8 |
| M11 | 130 | 131 | 86 | 86 | 25 | 25 | 87.8 | 88.8 | 17.2 | 17.2 |
| M12 | 146 | 122 | 98 | 96 | 22 | 24 | 104.4 | 78.8 | 19.4 | 19.2 |
| M13 | 132 | 127 | 172 | 170 | 27 | 29 | 70.6 | 64 | 34.4 | 34 |
| M14 | 190 | 178 | 103 | 92 | 34 | 36 | 135.4 | 123.6 | 20.6 | 18.4 |
| M15 | 98 | 206 | 73 | 104 | 18 | 20 | 65.4 | 165.2 | 14.6 | 20.2 |
| M16 | 145 | 123 | 89 | 83 | 25 | 27 | 102.2 | 79.4 | 17.8 | 16.6 |
| M17 | 193 | 190 | 91 | 98 | 27 | 37 | 147.8 | 133.4 | 18.2 | 19.6 |
| M18 | 114 | 104 | 99 | 101 | 26 | 28 | 68.2 | 55.8 | 19.8 | 20.2 |
| M19 | 90 | 98 | 78 | 100 | 19 | 15 | 55.4 | 63 | 15.6 | 20 |
| M20 | 201 | 191 | 105 | 96 | 41 | 38 | 139 | 133.8 | 21 | 19.2 |
| M21 | 116 | 95 | 104 | 84 | 23 | 35 | 72.2 | 78.2 | 20.8 | 16.6 |
| M22 | 193 | 190 | 91 | 98 | 37 | 42 | 137.8 | 128.4 | 18.2 | 19.6 |
| M23 | 264 | 170 | 193 | 106 | 42 | 44 | 183.4 | 104.8 | 38.6 | 21.2 |
| M24 | 135 | 128 | 98 | 103 | 38 | 40 | 77.4 | 67.4 | 19.6 | 20.6 |
| M25 | 120 | 136 | 80 | 82 | 19 | 32 | 85 | 87.6 | 16 | 16.4 |
| M26 | 129 | 117 | 90 | 76 | 28 | 30 | 83 | 71.8 | 18 | 15.2 |
| M27 | 265 | 123 | 148 | 84 | 40 | 43 | 195.4 | 63.2 | 29.6 | 16.6 |
| M28 | 157 | 157 | 113 | 112 | 33 | 33 | 101.4 | 101.6 | 22.6 | 22.4 |
| M29 | 152 | 133 | 107 | 89 | 31 | 35 | 99.6 | 80.2 | 21.4 | 17.8 |
| M30 | 194 | 198 | 105 | 99 | 38 | 40 | 135 | 138.2 | 21 | 18.2 |
| M31 | 184 | 178 | 87 | 81 | 28 | 32 | 138.6 | 129.8 | 17.4 | 16.6 |
