# Supplementary material for: Intermittent fasting positively modulates human gut microbial diversity and ameliorates blood lipid profile
Source: Front Microbiol. 2022 Aug 23;13:922727. doi: 10.3389/fmicb.2022.922727 (PMC9445987; doi:10.3389/fmicb.2022.922727)
Supplement: Supplementary Table 2 — Impact of intermittent fasting of serum lipid profile of female participants. [file Table_2.docx]

| Sample ID | Before TC (mg/dL) | After TC  (mg/dL) | before TG  (mg/dL) | After TG  (mg/dL) | Before HDL  (mg/dL) | After HDL  (mg/dL) | Before LDL  (mg/dL) | After LDL  (mg/dL) | Before vLDL  (mg/dL) | After vLDL  (mg/dL) |
| --- | --- | --- | --- | --- | --- | --- | --- | --- | --- | --- |
| F1 | 191 | 184 | 68 | 66 | 38 | 39 | 139.4 | 131.8 | 13.20 | 13.60 |
| F2 | 130 | 140 | 95 | 102 | 26 | 29 | 85 | 90.6 | 19.0 | 20.4 |
| F3 | 200 | 149 | 189 | 141 | 42 | 32 | 120.2 | 88.8 | 37.8 | 27.4 |
| F4 | 170 | 118 | 160 | 88 | 32 | 47 | 106 | 53.4 | 32.0 | 17.6 |
| F6 | 118 | 107 | 98 | 92 | 23 | 24 | 75.4 | 64.6 | 19.6 | 18.2 |
| F7 | 160 | 137 | 93 | 84 | 11 | 15 | 174.4 | 105.2 | 19.8 | 16.8 |
| F9 | 147 | 145 | 107 | 105 | 22 | 22 | 103.6 | 102 | 21.4 | 21.0 |
| F11 | 145 | 152 | 98 | 93 | 26 | 28 | 99.4 | 105.4 | 19.6 | 19.8 |
| F12 | 108 | 102 | 62 | 63 | 22 | 23 | 73.6 | 66.4 | 12.4 | 12.6 |
| F14 | 144 | 144 | 115 | 95 | 38 | 55 | 83 | 70 | 23.0 | 19.0 |
| F20 | 272 | 199 | 115 | 100 | 42 | 43 | 207 | 136 | 23.0 | 20.0 |
| F23 | 74 | 100 | 55 | 93 | 20 | 57 | 43 | 24.4 | 11.0 | 18.4 |
| F25 | 176 | 201 | 95 | 107 | 35 | 36 | 122 | 143.6 | 19.0 | 21.0 |
| F29 | 184 | 169 | 103 | 98 | 32 | 34 | 131.4 | 115.4 | 20.6 | 19.0 |
