# Supplementary material for: Intermittent fasting positively modulates human gut microbial diversity and ameliorates blood lipid profile
Source: Front Microbiol. 2022 Aug 23;13:922727. doi: 10.3389/fmicb.2022.922727 (PMC9445987; doi:10.3389/fmicb.2022.922727)
Supplement: Supplementary Table 3 — Impact of intermittent fasting of gut microbiota at phylum level of participants. [file Table_3.docx]

| **Phyla** | **Female** | | | | | | **Male** | | | |
| --- | --- | --- | --- | --- | --- | --- | --- | --- | --- | --- |
|  | **Overweight/obese** | | **Normal** | | **underweight** | | **Normal** | | **underweight** | |
|  | Before Fasting | After Fasting | Before Fasting | After Fasting | Before Fasting | After Fasting | Before Fasting | After Fasting | Before Fasting | After Fasting |
| ***Firmicutes*** | 60.47  ±  3.02 | 54.43  ±  3.02 | 60.47  ±  1.67 | 63.81  ±  1.67 | 86.91  ±  1.18 | 84.54  ±  1.18 | 69.95  ±  14.76 | 40.42  ±  14.76 | 26.37  ±  16.27 | 58.91  ±  16.27 |
| ***Bacteroidetes*** | 22.57  ±  5.50 | 11.57  ±  5.50 | 22.57  ±  2.58 | 17.41  ±  2.58 | 3.86  ±  0.84 | 2.18  ±  0.84 | 8.87  ±  5.47 | 19.81  ±  5.47 | 0.11  ±  1.10 | 2.31  ±  1.10 |
| ***Actinobacteria*** | 12.12  ±  0.43 | 11.26  ±  0.43 | 12.12  ±  2.61 | 6.90  ±  2.61 | 5.53  ±  1.70 | 8.92  ±  1.70 | 19.72  ±  6.65 | 6.43  ±  6.65 | 0.19±  18.48 | 37.14  ±  18.48 |
| ***Proteobacteria*** | 4.17  ±  9.22 | 22.61  ±  9.22 | 4.17  ±  0.68 | 5.52  ±  0.68 | 1.57  ±  0.69 | 2.95  ±  0.69 | 0.95  ±  11.92 | 24.78  ±  11.92 | 73.28±  35.86 | 1.56  ±  35.86 |
| ***Cyanobacteria*** | 0.45  ±  0.21 | 0.04  ±  0.21 | 0.45  ±  0.99 | 2.44  ±  0.99 | 1.20  ±  0.56 | 0.08  ±  0.56 | 0.01  ±  0.00 | 0.00  ±  0.00 | 0.01  ±  0.00 | 0.01  ±  0.00 |
| ***Verrucomicrobia*** | 0.07  ±  0.02 | 0.03  ±  0.02 | 0.07  ±  1.67 | 3.41  ±  1.67 | 0.02  ±  0.01 | 0.01  ±  0.01 | 0.21  ±  3.88 | 7.98  ±  3.88 | 0.01  ±  0.00 | 0.01  ±  0.00 |
| ***Spirochaetes*** | 0.05  ±  0.02 | 0.02  ±  0.02 | 0.05  ±  0.02 | 0.09  ±  0.02 | 0.80  ±  0.24 | 0.32  ±  0.24 | 0.22  ±  0.05 | 0.32  ±  0.05 | 0.00  ±  0.00 | 0.01  ±  0.00 |
| ***Lentisphaerae*** | 0.01  ±  0.01 | 0.00  ±  0.01 | 0.01  ±  0.06 | 0.14  ±  0.06 | 0.01  ±  0.00 | 0.00  ±  0.00 | 0.00  ±  0.09 | 0.17  ±  0.09 | 0.00  ±  0.00 | 0.00  ±  0.00 |
| ***Tenericutes*** | 0.00  ±  0.00 | 0.00  ±  0.00 | 0.00  ±  0.11 | 0.23  ±  0.11 | 0.06  ±  0.45 | 0.96  ±  0.45 | 0.03  ±  0.01 | 0.04  ±  0.01 | 0.00  ±  0.00 | 0.00  ±  0.00 |
