# Supplementary material for: Intermittent fasting positively modulates human gut microbial diversity and ameliorates blood lipid profile
Source: Front Microbiol. 2022 Aug 23;13:922727. doi: 10.3389/fmicb.2022.922727 (PMC9445987; doi:10.3389/fmicb.2022.922727)
Supplement: Supplementary Table 4 — Impact of intermittent fasting of gut microbiota at genera level of obese female participants. [file Table_4.docx]

| Before Fasting | | | After Fasting | | |
| --- | --- | --- | --- | --- | --- |
| Bacterial Genera | No of OUTs | %age | *Bacterial Genera* | No of OTUs | % age |
| *Roseburia faecis* | 59741 | 15.34 | *Shigella sonnei* | 40848 | 16.35 |
| *Prevotella copri* | 45041 | 11.57 | *Clostridium spp.* | 35957 | 14.40 |
| *Bifidobacterium adolescentis* | 32749 | 8.41 | *Clostridium perfringens* | 26812 | 10.73 |
| *Faecalibacterium spp.* | 22428 | 5.76 | *Prevotella copri* | 17514 | 7.01 |
| *Ruminococcaceae* | 18775 | 4.82 | *Bifidobacterium adolescentis* | 14481 | 5.80 |
| *Faecalibacterium prausnitzii* | 11083 | 2.85 | *Subdoligranulum spp.* | 13642 | 5.46 |
| *Paraprevotella* | 10235 | 2.63 | *Campylobacter spp.* | 7364 | 2.95 |
| *Dialister succinatiphilus* | 9265 | 2.38 | *Ruminococcaceae* | 7264 | 2.91 |
| *Eubacterium rectale* | 8688 | 2.23 | *Coriobacteriaceae* | 6083 | 2.44 |
| *Clostridiales* | 7562 | 1.94 | *Megasphaera elsdenii* | 5410 | 2.17 |
| *Mitsuokella multacida* | 7444 | 1.91 | *Dialister succinatiphilus* | 4910 | 1.97 |
| *Oscillospira spp.* | 7166 | 1.84 | *Faecalibacterium spp.* | 4351 | 1.74 |
| *Clostridium* | 7021 | 1.80 | *Faecalibacterium prausnitzii* | 4140 | 1.66 |
| *Roseburia* | 6865 | 1.76 | *Intestinibacter clostridium bartlettii* | 4067 | 1.63 |
| *Prevotellaceae* | 6262 | 1.61 | *Prevotella* | 4039 | 1.62 |
| *Prevotella* | 5896 | 1.51 | *Paraprevotella* | 3296 | 1.32 |
| *Dorea spp.* | 5635 | 1.45 | *Collinsella aerofaciens* | 3188 | 1.28 |
| *Clostridiaceae* | 5374 | 1.38 | *Clostridium disporicum* | 2530 | 1.01 |
| *Ruminococcus spp.* | 4824 | 1.24 | *Roseburia faecis* | 2430 | 0.97 |
| *Olsenella sp.* | 4707 | 1.21 | *Subdoligranulum* | 2288 | 0.92 |
| *Subdoligranulum spp.* | 4182 | 1.07 | *Desulfovibrio spp.* | 1743 | 0.70 |
| *Prevotella stercorea* | 4102 | 1.05 | *Prevotella sp.* | 1619 | 0.65 |
| *Clostridium spp.* | 3871 | 0.99 | *Lactobacillus ruminis* | 1530 | 0.61 |
| *Comamonas kerstersii* | 3548 | 0.91 | *Clostridiales* | 1354 | 0.54 |
| *Cytophagales* | 3533 | 0.91 | *Dorea spp.* | 1288 | 0.52 |
| *Sutterella wadsworthensis* | 3474 | 0.89 | *Serratia marcescens* | 1268 | 0.51 |
| *Catenibacterium mitsuokai* | 2932 | 0.75 | *Haemophilus parainfluenzae* | 1215 | 0.49 |
| *Blautia spp.* | 2921 | 0.75 | *Catenibacterium mitsuokai* | 1167 | 0.47 |
| *Coriobacteriaceae* | 2734 | 0.70 | *Streptococcus salivarius* | 1157 | 0.46 |
| *Oscillospira* | 2634 | 0.68 | *Enterobacter hormaechei* | 1113 | 0.45 |
| *Bacteroidales* | 2524 | 0.65 | *Blautia spp.* | 1060 | 0.42 |
| *Eubacteriaceae* | 2486 | 0.64 | *Eubacterium* | 1013 | 0.41 |
| *Paludibacter spp.* | 2465 | 0.63 | *Prevotellaceae* | 1013 | 0.41 |
| *Desulfovibrio spp.* | 2432 | 0.62 | *Olsenella sp.* | 1012 | 0.41 |
| *Porphyromonadaceae* | 2417 | 0.62 | *Slackia spp.* | 998 | 0.40 |
| *Mitsuokella jalaludinii* | 2020 | 0.52 | *Clostridium* | 995 | 0.40 |
| *Prevotella sp.* | 1996 | 0.51 | *Mitsuokella jalaludinii* | 966 | 0.39 |
| *Lactobacillus ruminis* | 1811 | 0.47 | *Acidaminococcus fermentans* | 866 | 0.35 |
| *Olsenella spp.* | 1806 | 0.46 | *Olsenella spp.* | 834 | 0.33 |
| *Gloeobacterales* | 1765 | 0.45 | *Mitsuokella* | 744 | 0.30 |
| *Eubacterium* | 1746 | 0.45 | *Mitsuokella multacida* | 720 | 0.29 |
| *Faecalibacterium* | 1669 | 0.43 | *Serratia nematodiphila* | 708 | 0.28 |
| *Megasphaera elsdenii* | 1603 | 0.41 | *Faecalibacterium* | 679 | 0.27 |
| *Ruminococcus callidus* | 1526 | 0.39 | *Eubacteriaceae* | 605 | 0.24 |
| *Roseburia inulinivorans* | 1461 | 0.38 | *Ruminococcus spp.* | 570 | 0.23 |
| *Kopriimonadaceae* | 1253 | 0.32 | *Oscillospira spp.* | 553 | 0.22 |
| *Lachnospiraceae* | 1102 | 0.28 | *Porphyromonadaceae* | 424 | 0.17 |
| *Blautia ruminococcus obeum* | 1065 | 0.27 | *Prevotella stercorea* | 424 | 0.17 |
| *Coprococcus eutactus* | 1063 | 0.27 | *Eubacterium rectale* | 422 | 0.17 |
| *Ruminococcus* | 1053 | 0.27 | *Clostridiaceae* | 413 | 0.17 |
| *Blautia* | 1030 | 0.26 | *Holdemanella eubacterium biforme* | 396 | 0.16 |
| *Dorea formicigenerans* | 968 | 0.25 | *Sphingomonas caulobacter leidyia* | 376 | 0.15 |
| *Coprococcus spp.* | 965 | 0.25 | *Dorea formicigenerans* | 336 | 0.13 |
| *Paludibacter* | 962 | 0.25 | *Ruminococcus* | 287 | 0.11 |
| *Coprococcus* | 954 | 0.25 | *Clostridium sp.* | 273 | 0.11 |
| *Oribacterium* | 825 | 0.21 | *Collinsella sp.* | 256 | 0.10 |
| *Haemophilus parainfluenzae* | 819 | 0.21 |  |  |  |
| *Enterobacteriales* | 812 | 0.21 |  |  |  |
| *Collinsella aerofaciens* | 792 | 0.20 |  |  |  |
| *Actinobacteria* | 763 | 0.20 |  |  |  |
| *Coprococcus catus* | 759 | 0.19 |  |  |  |
| *Intestinibacter clostridium bartlettii* | 730 | 0.19 |  |  |  |
| *Sutterella* | 713 | 0.18 |  |  |  |
| *Holdemanella eubacterium biforme* | 707 | 0.18 |  |  |  |
| *Roseburia intestinalis* | 688 | 0.18 |  |  |  |
| *Shigella sonnei* | 682 | 0.18 |  |  |  |
| *Alistipes sp.* | 669 | 0.17 |  |  |  |
| *Lachnoclostridium* | 646 | 0.17 |  |  |  |
| *Clostridium sp.* | 622 | 0.16 |  |  |  |
| *Mitsuokella* | 562 | 0.14 |  |  |  |
| *Olsenella* | 545 | 0.14 |  |  |  |
| *Ruminococcus sp.* | 538 | 0.14 |  |  |  |
| *Bifidobacterium* | 529 | 0.14 |  |  |  |
| *Roseburia hominis* | 513 | 0.13 |  |  |  |
| *Subdoligranulum* | 496 | 0.13 |  |  |  |
| *Slackia spp.* | 486 | 0.12 |  |  |  |
| *Bifidobacterium thermophilum* | 484 | 0.12 |  |  |  |
| *Clostridium disporicum* | 479 | 0.12 |  |  |  |
| *Sutterella sp.* | 460 | 0.12 |  |  |  |
| *Senegalimassilia anaerobia* | 410 | 0.11 |  |  |  |
| *Lactobacillus spp.* | 401 | 0.10 |  |  |  |
| *Fusicatenibacter saccharivorans* | 400 | 0.10 |  |  |  |
