# Supplementary material for: Intermittent fasting positively modulates human gut microbial diversity and ameliorates blood lipid profile
Source: Front Microbiol. 2022 Aug 23;13:922727. doi: 10.3389/fmicb.2022.922727 (PMC9445987; doi:10.3389/fmicb.2022.922727)
Supplement: Supplementary Table 5 — Impact of intermittent fasting of gut microbiota at genera level of normal weight female participants. [file Table_5.docx]

| Bacterial Genera | Before fasting | | Bacterial Genera | Normal (After) | |
| --- | --- | --- | --- | --- | --- |
|  | OTUs count | %age |  | OTUs count | %age |
| *Bacillaceae* | 779 | 0.21 | *Actinobacteria* | 6119 | 1.56 |
| *Bacteroidales* | 13239 | 3.57 | *Akkermansia spp.* | 13299 | 3.40 |
| *Bacteroides vulgatus* | 6007 | 1.62 | *Alistipes* | 1109 | 0.28 |
| *Bifidobacterium adolescentis* | 20806 | 5.61 | *Alistipes finegoldii* | 1731 | 0.44 |
| *Bifidobacterium thermophilum* | 605 | 0.16 | *Alistipes putredinis* | 3369 | 0.86 |
| *Blautia spp.* | 700 | 0.18 | *Alistipes senegalensis* | 458 | 0.11 |
| *Butyrivibrio crossotus* | 2891 | 0.77 | *Alistipes shahii* | 4724 | 1.20 |
| *Campylobacter spp.* | 62797 | 16.94 | *Alistipes sp.* | 2790 | 0.71 |
| *Campylobacter upsaliensis* | 5662 | 1.52 | *Bacillales* | 541 | 0.13 |
| *Catenibacterium mitsuokai* | 2219 | 0.59 | *Bacteroidales* | 587 | 0.15 |
| *Clostridiaceae* | 5886 | 1.58 | *Bacteroides cellulosilyticus* | 2042 | 0.52 |
| *Clostridiales* | 26967 | 7.27 | *Bacteroides clarus* | 618 | 0.15 |
| *Clostridium* | 874 | 0.23 | *Bacteroides fragilis* | 5826 | 1.49 |
| *Clostridium disporicum* | 639 | 0.17 | *Bacteroides ovatus* | 1899 | 0.48 |
| *Clostridium sp.* | 1402 | 0.37 | *Bacteroides salyersiae* | 677 | 0.17 |
| *Clostridium spp.* | 4532 | 1.22 | *Bacteroides uniformis* | 2333 | 0.59 |
| *Collinsella aerofaciens* | 699 | 0.18 | *Bacteroides vulgatus* | 2370 | 0.60 |
| *Cytophagales* | 10055 | 2.71 | *Barnesiella* | 602 | 0.15 |
| *Desulfovibrio spp.* | 1349 | 0.36 | *Barnesiella intestinihominis* | 21251 | 5.43 |
| *Dialister succinatiphilus* | 12298 | 3.31 | *Bifidobacterium adolescentis* | 14635 | 3.74 |
| *Dorea spp.* | 1590 | 0.42 | *Bifidobacterium catenulatum* | 915 | 0.23 |
| *Erysipelotrichales* | 1910 | 0.51 | *Bifidobacterium merycicum* | 508 | 0.12 |
| *Eubacteriaceae* | 7424 | 2.00 | *Bifidobacterium thermophilum* | 2206 | 0.56 |
| *Eubacterium* | 402 | 0.10 | *Blautia ruminococcus obeum* | 592 | 0.15 |
| *Eubacterium rectale* | 395 | 0.10 | *Blautia spp.* | 882 | 0.22 |
| *Faecalibacterium* | 877 | 0.23 | *Butyricimonas virosa* | 669 | 0.17 |
| *Faecalibacterium prausnitzii* | 5646 | 1.52 | *Campylobacter spp.* | 488 | 0.12 |
| *Faecalibacterium spp.* | 5258 | 1.41 | *Catenibacterium mitsuokai* | 1370 | 0.35 |
| *Flavobacteriia* | 414 | 0.11 | *Clostridiaceae* | 10026 | 2.56 |
| *Holdemanella eubacterium biforme* | 596 | 0.16 | *Clostridiales* | 6899 | 1.76 |
| *Intestinibacter clostridium bartlettii* | 416 | 0.11 | *Clostridium* | 1218 | 0.31 |
| *Lachnoclostridium* | 411 | 0.11 | *Clostridium disporicum* | 930 | 0.23 |
| *Lachnospiraceae* | 3025 | 0.81 | *Clostridium perfringens* | 550 | 0.14 |
| *Lactobacillales* | 1361 | 0.36 | *Clostridium sp.* | 393 | 0.10 |
| *Lactobacillus ruminis* | 2099 | 0.56 | *Clostridium spp.* | 20882 | 5.34 |
| *Megasphaera* | 442 | 0.11 | *Collinsella aerofaciens* | 748 | 0.19 |
| *Megasphaera elsdenii* | 18060 | 4.87 | *Coriobacteriaceae* | 541 | 0.13 |
| *Mitsuokella jalaludinii* | 1454 | 0.39 | *Cytophagales* | 568 | 0.14 |
| *Mitsuokella multacida* | 1043 | 0.28 | *Dialister succinatiphilus* | 422 | 0.10 |
| *Opitutae* | 504 | 0.13 | *Dorea spp.* | 988 | 0.25 |
| *Opitutales* | 2246 | 0.60 | *Enterobacter hormaechei* | 483 | 0.12 |
| *Oscillospira* | 4381 | 1.18 | *Enterobacteriales* | 963 | 0.24 |
| *Oscillospira spp.* | 11563 | 3.11 | *Entomoplasmatales* | 904 | 0.23 |
| *Paludibacter* | 1894 | 0.51 | *Erysipelotrichaceae* | 2458 | 0.62 |
| *Paludibacter spp.* | 2941 | 0.79 | *Erysipelotrichales* | 13694 | 3.50 |
| *Paraprevotella* | 2167 | 0.58 | *Eubacteriaceae* | 42638 | 10.90 |
| *Porphyromonadaceae* | 2841 | 0.76 | *Eubacterium* | 1044 | 0.26 |
| *Prevotella* | 823 | 0.22 | *Eubacterium rectale* | 709 | 0.18 |
| *Prevotella copri* | 5171 | 1.39 | *Faecalibacterium* | 6367 | 1.62 |
| *Prevotella sp.* | 729 | 0.19 | *Faecalibacterium prausnitzii* | 8369 | 2.14 |
| *Prevotellaceae* | 5305 | 1.43 | *Faecalibacterium spp.* | 7373 | 1.88 |
| *Puniceicoccales* | 26804 | 7.23 | *Gloeobacterales* | 9022 | 2.30 |
| *Rhodospirillales* | 15096 | 4.07 | *Halospirulina sp.* | 501 | 0.12 |
| *Robinsoniella* | 708 | 0.19 | *Holdemanella eubacterium biforme* | 1517 | 0.38 |
| *Roseburia faecis* | 1907 | 0.51 | *Intestinibacter clostridium bartlettii* | 4124 | 1.05 |
| *Ruminococcaceae* | 7438 | 2.00 | *Kopriimonadaceae* | 418 | 0.10 |
| *Ruminococcus spp.* | 2064 | 0.55 | *Kopriimonadales* | 581 | 0.14 |
| *Shigella sonnei* | 1515 | 0.40 | *Lactobacillus ruminis* | 523 | 0.13 |
| *Sphingobacteriales* | 19572 | 5.28 | *Odoribacter splanchnicus* | 745 | 0.19 |
| *Spirochaetaceae* | 1175 | 0.31 | *Oscillospira* | 1143 | 0.29 |
| *Subdoligranulum spp.* | 2707 | 0.73 | *Oscillospira spp.* | 3182 | 0.81 |
| *Succinivibrio* | 785 | 0.21 | *Paludibacter* | 2287 | 0.58 |
| *Sutterella sp.* | 1647 | 0.44 | *Paludibacter spp.* | 4104 | 1.04 |
| *Victivallis vadensis* | 581 | 0.15 | *Parabacteroides distasonis* | 725 | 0.18 |
|  |  |  | *Parabacteroides merdae* | 1783 | 0.45 |
|  |  |  | *Phascolarctobacterium spp.* | 893 | 0.22 |
|  |  |  | *Porphyromonadaceae* | 3635 | 0.92 |
|  |  |  | *Pseudomonas trivialis* | 747 | 0.19 |
|  |  |  | *Roseburia faecis* | 2416 | 0.61 |
|  |  |  | *Ruminococcaceae* | 4807 | 1.22 |
|  |  |  | *Ruminococcus* | 1197 | 0.30 |
|  |  |  | *Ruminococcus callidus* | 536 | 0.13 |
|  |  |  | *Ruminococcus sp.* | 841 | 0.21 |
|  |  |  | *Ruminococcus spp.* | 49259 | 12.59 |
|  |  |  | *Serratia marcescens* | 885 | 0.22 |
|  |  |  | *Serratia nematodiphila* | 503 | 0.12 |
|  |  |  | *Shigella sonnei* | 15335 | 3.92 |
|  |  |  | *Streptococcus* | 12934 | 3.30 |
|  |  |  | *Streptococcus lutetiensis* | 8740 | 2.23 |
|  |  |  | *Streptococcus salivarius* | 2398 | 0.61 |
|  |  |  | *Subdoligranulum* | 1000 | 0.25 |
|  |  |  | *Subdoligranulum spp.* | 18847 | 4.82 |
|  |  |  | *Victivallis vadensis* | 532 | 0.13 |
