# Supplementary material for: Intermittent fasting positively modulates human gut microbial diversity and ameliorates blood lipid profile
Source: Front Microbiol. 2022 Aug 23;13:922727. doi: 10.3389/fmicb.2022.922727 (PMC9445987; doi:10.3389/fmicb.2022.922727)
Supplement: Supplementary Table 6 — Impact of intermittent fasting of gut microbiota at genera level of under-weight female participants. [file Table_6.docx]

| Before Fasting | | | | After Fasting | | |
| --- | --- | --- | --- | --- | --- | --- |
| Bacterial Genera | OTUs count | %age | | Bacterial Genera | OTUs count | %age |
| *Bacillaceae* | 280 | | 0.11 | *Bacillaceae* | 326 | 0.12 |
| *Bacteroidales* | 1929 | | 0.73 | *Bacteroidales* | 1149 | 0.44 |
| *Bifidobacterium adolescentis* | 9358 | | 3.56 | *Bifidobacterium adolescentis* | 9500 | 3.63 |
| *Bifidobacterium thermophilum* | 962 | | 0.37 | *Bifidobacterium catenulatum* | 558 | 0.21 |
| *Blautia spp.* | 1393 | | 0.53 | *Bifidobacterium merycicum* | 537 | 0.21 |
| *Campylobacter spp.* | 809 | | 0.31 | *Bifidobacterium thermophilum* | 2385 | 0.91 |
| *Catenibacterium mitsuokai* | 1248 | | 0.47 | *Blautia spp.* | 1485 | 0.57 |
| *Clostridiaceae* | 11120 | | 4.23 | *Bulleidia spp.* | 660 | 0.25 |
| *Clostridiales* | 11626 | | 4.42 | *Campylobacter spp.* | 435 | 0.17 |
| *Clostridium* | 4038 | | 1.53 | *Catenibacterium mitsuokai* | 8007 | 3.06 |
| *Clostridium disporicum* | 22715 | | 8.63 | *Clostridiaceae* | 9068 | 3.46 |
| *Clostridium perfringens* | 1439 | | 0.55 | *Clostridiales* | 5657 | 2.16 |
| *Clostridium sp.* | 1969 | | 0.75 | *Clostridium* | 1952 | 0.75 |
| *Clostridium spp.* | 47395 | | 18.01 | *Clostridium disporicum* | 8524 | 3.26 |
| *Collinsella aerofaciens* | 1408 | | 0.54 | *Clostridium perfringens* | 939 | 0.36 |
| *Coprococcus eutactus* | 853 | | 0.32 | *Clostridium sp.* | 608 | 0.23 |
| *Coriobacteriaceae* | 927 | | 0.35 | *Clostridium spp.* | 63451 | 24.23 |
| *Cytophagales* | 661 | | 0.25 | *Collinsella aerofaciens* | 5482 | 2.09 |
| *Dialister succinatiphilus* | 2892 | | 1.10 | *Coriobacteriaceae* | 1745 | 0.67 |
| *Dorea spp.* | 1605 | | 0.61 | *Dialister succinatiphilus* | 2401 | 0.92 |
| *Enterobacter hormaechei* | 304 | | 0.12 | *Dorea spp.* | 5138 | 1.96 |
| *Erysipelotrichaceae* | 17873 | | 6.79 | *Enterobacter hormaechei* | 297 | 0.11 |
| *Erysipelotrichales* | 7752 | | 2.95 | *Enterococcus faecalis* | 1162 | 0.44 |
| *Eubacteriaceae* | 9528 | | 3.62 | *Entomoplasmatales* | 2472 | 0.94 |
| *Eubacterium* | 851 | | 0.32 | *Erysipelotrichaceae* | 3611 | 1.38 |
| *Eubacterium rectale* | 463 | | 0.18 | *Erysipelotrichales* | 11765 | 4.49 |
| *Faecalibacterium* | 877 | | 0.33 | *Eubacteriaceae* | 18242 | 6.97 |
| *Faecalibacterium prausnitzii* | 1820 | | 0.69 | *Eubacterium* | 366 | 0.14 |
| *Faecalibacterium spp.* | 2999 | | 1.14 | *Eubacterium rectale* | 515 | 0.20 |
| *Gloeobacterales* | 3149 | | 1.20 | *Faecalibacterium* | 356 | 0.14 |
| *Holdemanella eubacterium biforme* | 344 | | 0.13 | *Faecalibacterium prausnitzii* | 810 | 0.31 |
| *Intestinibacter clostridium bartlettii* | 7424 | | 2.82 | *Faecalibacterium spp.* | 1268 | 0.48 |
| *Lachnoclostridium* | 1212 | | 0.46 | *Granulicatella elegans* | 449 | 0.17 |
| *Lachnospiraceae* | 324 | | 0.12 | *Holdemanella eubacterium biforme* | 1025 | 0.39 |
| *Lactobacillales* | 350 | | 0.13 | *Intestinibacter clostridium bartlettii* | 8264 | 3.16 |
| *Lactobacillus ruminis* | 690 | | 0.26 | *Lachnoclostridium* | 1040 | 0.40 |
| *Megasphaera elsdenii* | 842 | | 0.32 | *Lactobacillus ruminis* | 303 | 0.12 |
| *Mitsuokella jalaludinii* | 586 | | 0.22 | *Lactococcus garvieae* | 1724 | 0.66 |
| *Mitsuokella multacida* | 419 | | 0.16 | *Oscillospira* | 3154 | 1.20 |
| *Oscillospira* | 3158 | | 1.20 | *Oscillospira spp.* | 6901 | 2.64 |
| *Oscillospira spp.* | 7703 | | 2.93 | *Paludibacter* | 804 | 0.31 |
| *Paludibacter* | 996 | | 0.38 | *Paludibacter spp.* | 1003 | 0.38 |
| *Paludibacter spp.* | 1312 | | 0.50 | *Peptococcus* | 3030 | 1.16 |
| *Paraprevotella* | 702 | | 0.27 | *Porphyromonadaceae* | 928 | 0.35 |
| *Peptococcus* | 685 | | 0.26 | *Prevotella copri* | 738 | 0.28 |
| *Porphyromonadaceae* | 995 | | 0.38 | *Rhodospirillales* | 264 | 0.10 |
| *Prevotella copri* | 2046 | | 0.78 | *Roseburia* | 265 | 0.10 |
| *Prevotella sp.* | 328 | | 0.12 | *Roseburia faecis* | 1734 | 0.66 |
| *Pseudomonas trivialis* | 356 | | 0.14 | *Ruminococcaceae* | 8591 | 3.28 |
| *Rhodospirillales* | 357 | | 0.14 | *Ruminococcus* | 854 | 0.33 |
| *Roseburia* | 1457 | | 0.55 | *Ruminococcus sp.* | 5103 | 1.95 |
| *Roseburia faecis* | 1964 | | 0.75 | *Ruminococcus spp.* | 15218 | 5.81 |
| *Ruminococcaceae* | 19292 | | 7.33 | *Senegalimassilia anaerobia* | 1485 | 0.57 |
| *Ruminococcus* | 559 | | 0.21 | *Serratia marcescens* | 337 | 0.13 |
| *Ruminococcus callidus* | 1243 | | 0.47 | *Shigella sonnei* | 5413 | 2.07 |
| *Ruminococcus sp.* | 786 | | 0.30 | *Slackia spp.* | 595 | 0.23 |
| *Ruminococcus spp.* | 19039 | | 7.23 | *Spirochaetaceae* | 806 | 0.31 |
| *Senegalimassilia anaerobia* | 499 | | 0.19 | *Streptococcus* | 319 | 0.12 |
| *Shigella sonnei* | 992 | | 0.38 | *Streptococcus salivarius* | 1579 | 0.60 |
| *Slackia spp.* | 277 | | 0.11 | *Subdoligranulum* | 521 | 0.20 |
| *Spirochaetaceae* | 2052 | | 0.78 | *Subdoligranulum spp.* | 9418 | 3.60 |
| *Streptococcus salivarius* | 779 | | 0.30 |  |  |  |
| *Subdoligranulum spp.* | 3112 | | 1.18 |  |  |  |
| *Turicibacter spp.* | 615 | | 0.23 |  |  |  |
