# Supplementary material for: Intermittent fasting positively modulates human gut microbial diversity and ameliorates blood lipid profile
Source: Front Microbiol. 2022 Aug 23;13:922727. doi: 10.3389/fmicb.2022.922727 (PMC9445987; doi:10.3389/fmicb.2022.922727)
Supplement: Supplementary Table 7 — Impact of intermittent fasting of gut microbiota at genera level of normal weight male participants. [file Table_7.docx]

| Before Fasting | | | After Fasting | | |
| --- | --- | --- | --- | --- | --- |
| Bacterial Genera | OTUs count | %age | Bacterial Genera | OTUs count | %age |
| *Ruminococcus* | 113699 | 35.46 | *Campylobacter spp.* | 62797 | 16.94 |
| *Bifidobacterium* | 39452 | 12.31 | *Clostridiales* | 26967 | 7.28 |
| *Eubacterium* | 23800 | 7.42 | *Puniceicoccales* | 26804 | 7.23 |
| *Clostridium* | 20308 | 6.33 | *Bifidobacterium adolescentis* | 20806 | 5.61 |
| *Oscillospira* | 16153 | 5.04 | *Sphingobacteriales* | 19572 | 5.28 |
| *Paludibacter* | 11893 | 3.71 | *Megasphaera elsdenii* | 18060 | 4.87 |
| *Prevotella* | 10071 | 3.14 | *Rhodospirillales* | 15096 | 4.07 |
| *Collinsella* | 9594 | 2.99 | *Bacteroidales* | 13239 | 3.57 |
| *Olsenella* | 8440 | 2.63 | *Dialister succinatiphilus* | 12298 | 3.32 |
| *Subdoligranulum* | 7821 | 2.44 | *Oscillospira spp.* | 11563 | 3.12 |
| *Coprococcus* | 7329 | 2.29 | *Cytophagales* | 10055 | 2.71 |
| *Dorea* | 6632 | 2.07 | *Ruminococcaceae* | 7438 | 2.01 |
| *Catenibacterium* | 5911 | 1.84 | *Eubacteriaceae* | 7424 | 2.00 |
| *Faecalibacterium* | 3436 | 1.07 | *Bacteroides vulgatus* | 6007 | 1.62 |
| *Megasphaera* | 3165 | 0.99 | *Clostridiaceae* | 5886 | 1.59 |
| *Senegalimassilia* | 2960 | 0.92 | *Campylobacter upsaliensis* | 5662 | 1.53 |
| *Bacteroides* | 2499 | 0.78 | *Faecalibacterium prausnitzii* | 5646 | 1.52 |
| *Ruminiclostridium* | 2113 | 0.66 | *Prevotellaceae* | 5305 | 1.43 |
| *Campylobacter* | 1814 | 0.57 | *Faecalibacterium spp.* | 5258 | 1.42 |
| *Turicibacter* | 1616 | 0.50 | *Prevotella copri* | 5171 | 1.40 |
| *Blautia* | 1525 | 0.48 | *Clostridium spp.* | 4532 | 1.22 |
| *Roseburia* | 1483 | 0.46 | *Oscillospira* | 4381 | 1.18 |
| *Dialister* | 1422 | 0.44 | *Lachnospiraceae* | 3025 | 0.82 |
| *Alloprevotella* | 1393 | 0.43 | *Paludibacter spp.* | 2941 | 0.79 |
| *Rikenella* | 1248 | 0.39 | *Butyrivibrio crossotus* | 2891 | 0.78 |
| *Holdemanella* | 1088 | 0.34 | *Porphyromonadaceae* | 2841 | 0.77 |
| *Denitrobacterium* | 1082 | 0.34 | *Subdoligranulum spp.* | 2707 | 0.73 |
| *Slackia* | 1007 | 0.31 | *Opitutales* | 2246 | 0.61 |
| *Lactobacillus* | 924 | 0.29 | *Catenibacterium mitsuokai* | 2219 | 0.60 |
| *Mitsuokella* | 748 | 0.23 | *Paraprevotella* | 2167 | 0.58 |
| *Spirochaeta* | 717 | 0.22 | *Lactobacillus ruminis* | 2099 | 0.57 |
| *Robinsoniella* | 665 | 0.21 | *Ruminococcus spp.* | 2064 | 0.56 |
| *Cerasicoccus* | 598 | 0.19 | *Erysipelotrichales* | 1910 | 0.52 |
| *Sporobacter* | 478 | 0.15 | *Roseburia faecis* | 1907 | 0.51 |
| *Bulleidia* | 410 | 0.13 | *Paludibacter* | 1894 | 0.51 |
| *Paraprevotella* | 392 | 0.12 | *Sutterella sp.* | 1647 | 0.44 |
| *Cytophaga* | 390 | 0.12 | *Dorea spp.* | 1590 | 0.43 |
| *Parasporobacterium* | 384 | 0.12 | *Shigella sonnei* | 1515 | 0.41 |
| *Candidatus soleaferrea* | 333 | 0.10 | *Mitsuokella jalaludinii* | 1454 | 0.39 |
|  |  |  | *Clostridium sp.* | 1402 | 0.38 |
|  |  |  | *Lactobacillales* | 1361 | 0.37 |
|  |  |  | *Desulfovibrio spp.* | 1349 | 0.36 |
|  |  |  | *Spirochaetaceae* | 1175 | 0.32 |
|  |  |  | *Mitsuokella multacida* | 1043 | 0.28 |
|  |  |  | *Faecalibacterium* | 877 | 0.24 |
|  |  |  | *Clostridium* | 874 | 0.24 |
|  |  |  | *Prevotella* | 823 | 0.22 |
|  |  |  | *Succinivibrio* | 785 | 0.21 |
|  |  |  | *Bacillaceae* | 779 | 0.21 |
|  |  |  | *Prevotella sp.* | 729 | 0.20 |
|  |  |  | *Robinsoniella* | 708 | 0.19 |
|  |  |  | *Blautia spp.* | 700 | 0.19 |
|  |  |  | *Collinsella aerofaciens* | 699 | 0.19 |
|  |  |  | *Clostridium disporicum* | 639 | 0.17 |
|  |  |  | *Bifidobacterium thermophilum* | 605 | 0.16 |
|  |  |  | *Holdemanella eubacterium biforme* | 596 | 0.16 |
|  |  |  | *Victivallis vadensis* | 581 | 0.16 |
|  |  |  | *Opitutae* | 504 | 0.14 |
|  |  |  | *Megasphaera* | 442 | 0.12 |
|  |  |  | *Intestinibacter clostridium bartlettii* | 416 | 0.11 |
|  |  |  | *Flavobacteriia* | 414 | 0.11 |
|  |  |  | *Lachnoclostridium* | 411 | 0.11 |
|  |  |  | *Eubacterium* | 402 | 0.11 |
|  |  |  | *Eubacterium rectale* | 395 | 0.11 |
