# Supplementary material for: Intermittent fasting positively modulates human gut microbial diversity and ameliorates blood lipid profile
Source: Front Microbiol. 2022 Aug 23;13:922727. doi: 10.3389/fmicb.2022.922727 (PMC9445987; doi:10.3389/fmicb.2022.922727)
Supplement: Supplementary Table 8 — Impact of intermittent fasting of gut microbiota at genera level of under-weight male participants. [file Table_8.docx]

| Before Fasting | | | After Fasting | | |
| --- | --- | --- | --- | --- | --- |
| Bacterial Genera | OTUs count | %age | Bacteria Genera | OTUs count | %age |
| *Pseudomonas trivialis* | 291260 | 69.47 | *Bacteroidales* | 607 | 0.15 |
| *Brevibacillus limnophilus* | 68047 | 16.23 | *Bifidobacterium* | 1048 | 0.26 |
| *Lysinibacillus sphaericus* | 19423 | 4.63 | *Bifidobacterium adolescentis* | 114466 | 28.12 |
| *Bacillus sp.* | 15254 | 3.64 | *Bifidobacterium catenulatum* | 2346 | 0.58 |
| *Pseudomonas mendocina* | 4675 | 1.12 | *Bifidobacterium merycicum* | 1272 | 0.31 |
| *Pseudomonas sp.* | 3535 | 0.84 | *Bifidobacterium thermophilum* | 4683 | 1.15 |
| *Pseudomonas* | 1166 | 0.28 | *Blautia spp.* | 1673 | 0.41 |
| *Pseudomonas spp.* | 1045 | 0.25 | *Bulleidia spp.* | 2780 | 0.68 |
| *Paenibacillus spp.* | 862 | 0.21 | *Campylobacter spp.* | 1112 | 0.27 |
| *Enterobacter hormaechei* | 828 | 0.20 | *Catenibacterium* | 720 | 0.18 |
| *Pseudomonas syringae* | 717 | 0.17 | *Catenibacterium mitsuokai* | 22597 | 5.55 |
| *Shigella sonnei* | 691 | 0.16 | *Clostridiaceae* | 2283 | 0.56 |
| *Dialister succinatiphilus* | 639 | 0.15 | *Clostridiales* | 14571 | 3.58 |
| *Serratia marcescens* | 626 | 0.15 | *Clostridium* | 515 | 0.13 |
| *Campylobacter spp.* | 538 | 0.13 | *Clostridium spp.* | 2898 | 0.71 |
| *Ruminococcaceae* | 513 | 0.12 | *Collinsella aerofaciens* | 9819 | 2.41 |
| *Clostridium spp.* | 457 | 0.11 | *Coriobacteriaceae* | 7412 | 1.82 |
| *Bifidobacterium adolescentis* | 451 | 0.11 | *Dialister succinatiphilus* | 1575 | 0.39 |
|  |  |  | *Dorea formicigenerans* | 590 | 0.14 |
|  |  |  | *Dorea spp.* | 4377 | 1.08 |
|  |  |  | *Enterobacter hormaechei* | 1323 | 0.33 |
|  |  |  | *Eubacteriaceae* | 1854 | 0.46 |
|  |  |  | *Eubacterium* | 981 | 0.24 |
|  |  |  | *Eubacterium rectale* | 782 | 0.19 |
|  |  |  | *Eubacterium sp.* | 580 | 0.14 |
|  |  |  | *Faecalibacterium* | 659 | 0.16 |
|  |  |  | *Faecalibacterium prausnitzii* | 2226 | 0.55 |
|  |  |  | *Faecalibacterium spp.* | 1936 | 0.48 |
|  |  |  | *Holdemanella eubacterium biforme* | 3488 | 0.86 |
|  |  |  | *Lactobacillus ruminis* | 37245 | 9.15 |
|  |  |  | *Megasphaera elsdenii* | 2463 | 0.61 |
|  |  |  | *Olsenella* | 1230 | 0.30 |
|  |  |  | *Olsenella sp.* | 2513 | 0.62 |
|  |  |  | *Oscillospira spp.* | 2072 | 0.51 |
|  |  |  | *Paraprevotella* | 504 | 0.12 |
|  |  |  | *Porphyromonadaceae* | 672 | 0.17 |
|  |  |  | *Prevotella* | 635 | 0.16 |
|  |  |  | *Prevotella copri* | 3366 | 0.83 |
|  |  |  | *Prevotella sp.* | 2031 | 0.50 |
|  |  |  | *Prevotella stercorea* | 425 | 0.10 |
|  |  |  | *Roseburia faecis* | 3191 | 0.78 |
|  |  |  | *Roseburia inulinivorans* | 520 | 0.13 |
|  |  |  | *Ruminococcaceae* | 104532 | 25.68 |
|  |  |  | *Ruminococcus callidus* | 556 | 0.14 |
|  |  |  | *Ruminococcus spp.* | 1065 | 0.26 |
|  |  |  | *Senegalimassilia anaerobia* | 1725 | 0.42 |
|  |  |  | *Shigella sonnei* | 2275 | 0.56 |
|  |  |  | *Slackia spp.* | 3080 | 0.76 |
|  |  |  | *Streptococcus* | 537 | 0.13 |
|  |  |  | *Streptococcus salivarius* | 4410 | 1.08 |
|  |  |  | *Subdoligranulum* | 1007 | 0.25 |
|  |  |  | *Subdoligranulum spp.* | 8145 | 2.00 |
